# Memory Efficient PCA Methods for Large Group ICA

**DOI:** 10.3389/fnins.2016.00017

**Published:** 2016-02-02

**Authors:** Srinivas Rachakonda, Rogers F. Silva, Jingyu Liu, Vince D. Calhoun

**Affiliations:** ^1^The Mind Research Network and Lovelace Biomedical and Environmental Research InstituteAlbuquerque, NM, USA; ^2^Department of Electrical and Computer Engineering, The University of New MexicoAlbuquerque, NM, USA; ^3^Department of Computer Science, The University of New MexicoAlbuquerque, NM, USA

**Keywords:** group ICA, big data, PCA, subspace iteration, EVD, SVD, memory, power iteration

## Abstract

Principal component analysis (PCA) is widely used for data reduction in group independent component analysis (ICA) of fMRI data. Commonly, group-level PCA of temporally concatenated datasets is computed prior to ICA of the group principal components. This work focuses on reducing very high dimensional temporally concatenated datasets into its group PCA space. Existing randomized PCA methods can determine the PCA subspace with minimal memory requirements and, thus, are ideal for solving large PCA problems. Since the number of dataloads is not typically optimized, we extend one of these methods to compute PCA of very large datasets with a minimal number of dataloads. This method is coined multi power iteration (MPOWIT). The key idea behind MPOWIT is to estimate a subspace larger than the desired one, while checking for convergence of only the smaller subset of interest. The number of iterations is reduced considerably (as well as the number of dataloads), accelerating convergence without loss of accuracy. More importantly, in the proposed implementation of MPOWIT, the memory required for successful recovery of the group principal components becomes independent of the number of subjects analyzed. Highly efficient subsampled eigenvalue decomposition techniques are also introduced, furnishing excellent PCA subspace approximations that can be used for intelligent initialization of randomized methods such as MPOWIT. Together, these developments enable efficient estimation of accurate principal components, as we illustrate by solving a 1600-subject group-level PCA of fMRI with standard acquisition parameters, on a regular desktop computer with only 4 GB RAM, in just a few hours. MPOWIT is also highly scalable and could realistically solve group-level PCA of fMRI on thousands of subjects, or more, using standard hardware, limited only by time, not memory. Also, the MPOWIT algorithm is highly parallelizable, which would enable fast, distributed implementations ideal for big data analysis. Implications to other methods such as expectation maximization PCA (EM PCA) are also presented. Based on our results, general recommendations for efficient application of PCA methods are given according to problem size and available computational resources. MPOWIT and all other methods discussed here are implemented and readily available in the open source GIFT software.

## Introduction

Principal component analysis (PCA) is used as both a data reduction and de-noising method in group independent component analysis (ICA) (Calhoun et al., 2001; Beckmann and Smith, 2004; Calhoun and Adali, 2012). PCA is typically carried out by computing the eigenvalue decomposition (EVD) of the sample covariance matrix (*C*) or by using singular value decomposition (SVD) directly on the data. For large datasets, both EVD (plus computation of *C*) and SVD become computationally intensive in both memory and speed. For instance, group ICA is commonly used for functional magnetic resonance imaging (fMRI) studies. A typical fMRI study may collect image volumes from a single subject for about 20 min using TR = 1000 ms and 3 × 3 × 3 mm voxel resolution, resulting in approximately 53 × 63 × 46 × 1200 data points. To compute group PCA using the standard EVD of *C* approach on 100 subjects stacked in the temporal dimension (and only the nearly 70000 in-brain voxels) requires approximately 100 GB RAM and more than 16 h on a Linux server. Using the SVD approach would incur similar memory requirements as EVD (plus computation of *C*). In either case, the computational requirements can quickly become prohibitive, especially with the constant advance of imaging techniques [such as multi-band EPI sequences (Feinberg et al., 2010; Feinberg and Setsompop, 2013)] and a tendency to share data within the imaging community. This means very large size imaging data will become even more common for fMRI studies, encouraging the development of novel computational methods to face the upcoming challenges.

There are several methods to estimate dominant PCA components with minimal memory requirements, like sequential SVD, cascade recursive least squares (CRLS) PCA, and randomized PCA approaches, to name a few. Sequential or “online” SVD is usually applied in a streaming memory setting where the data streams over time and only a single pass over the datasets is possible. There exist algorithms (Brand, 2003; Li, 2004; Funk, 2006) which provide incremental SVD update and downdate capacity. However, principal components obtained with sequential SVD approaches are typically not as accurate as those from EVD of *C* and, therefore, sequential SVD approaches are considered not suitable for data reduction in group ICA analyses. CRLS PCA (Wang et al., 2006) uses a subspace deflation technique to extract dominant components of interest with limited training. The number of training epochs required is dependent on the data and, therefore, the CRLS PCA algorithm has slower performance in very large datasets and when higher model order (i.e., high number of components) needs to be estimated.

Randomized PCA methods are a class of algorithms that iteratively estimate the principal components from the data and are particularly useful when only a few components need to be estimated from very large datasets. They provide a much more efficient solution than the EVD approach, which always estimates the complete set of eigenvectors, many of which are eventually discarded for data reduction and de-noising purposes. Clearly, iterative approaches can make a much more intelligent use of the available computational resources. Some popular and upcoming randomized PCA approaches are: implicitly restarted Arnoldi iteration (IRAM; (Lehoucq and Sorensen, 1996)), power iteration (Recktenwald, 2000), subspace iteration (Rutishauser, 1970) expectation maximization PCA (EM PCA) (Roweis, 1997), and “Large PCA” (Halko et al., 2011a). IRAM as implemented in ARPACK (Lehoucq et al., 1998) requires that the sample covariance matrix be computed from the data and, thus, has higher computational demands on memory. Power iteration determines PCA components in a so-called “deflationary” mode (i.e., one at a time) and has very poor convergence properties when more than one component needs to be extracted from the data. Also, the error accumulates in subsequent estimations. Subspace iteration is a symmetric version of the power iteration method which extracts multiple components simultaneously from the data using explicit orthogonalization of the subspace in each iteration. EM PCA uses expectation and maximization steps to estimate multiple components simultaneously from the data. Both EM PCA and subspace iteration methods converge faster when only a few components are estimated from very large datasets and have slower convergence properties when a higher number of components needs to be estimated. More recently, Large PCA (Halko et al., 2011a) was proposed to evaluate the principal components from very large datasets. Large PCA is a randomized version of the block Lanczos method (Kuczynski and Wozniakowski, 1992) and is highly dependent on appropriate block size determination (typically large) in order to give accurate results with default settings.

In this paper, we show how to overcome the problem of slow convergence in subspace iteration when a high number of components is estimated by introducing a new approach, named multi power iteration (MPOWIT). Our approach takes into account the number of dataloads, which has often been overlooked in the development of randomized PCA methods. We also show that both subspace iteration and EM PCA methods converge to the same subspace in each iteration. Thus, the acceleration scheme we propose in MPOWIT can also be applied to EM PCA. In addition, we compare the performance of MPOWIT with existing PCA methods like EVD and Large PCA using real fMRI data from 1600 subjects with standard acquisition parameters. Moreover, acknowledging the recent popularization and promising developments in the area of multi-band EPI sequences (Feinberg and Setsompop, 2013), we provide performance assessments of the PCA methods discussed here in the case of hypothetical 5000-subject fMRI studies using TR = 200 ms, 2 × 2 × 2 mm voxel resolution, and 30 min-long sessions. Based on our current estimates, group-level PCA using our new randomized PCA approach and retaining 200 principal components in the subject-level PCA could be performed on such data in 40 h (nearly 36 h just loading the subject-level PCA results) using a Windows desktop with 4 GB RAM. Alternatively, the same analysis could be performed in just 2 h using a Linux server with 512 GB RAM (assuming the subject-level PCA results are kept in memory after their estimation). In either case, this is without the additional benefits of GPU acceleration or parallelization.

We provide descriptions of EVD, Subsampled PCA, Large PCA, MPOWIT and EM PCA in the Materials and Methods Section. The same section also includes a description of the datasets and experiments conducted for each PCA method. Experiments are performed on the real fMRI data. In the Results Section, we present our experimental results and compare the performance of MPOWIT with existing PCA methods. Finally, we discuss these results and draw conclusions based on the analyses we performed. Additional details are provided in the appendices, including a proof that EM PCA is equivalent to subspace iteration.

## Materials and methods

### Group ICA

**Figure 1:**
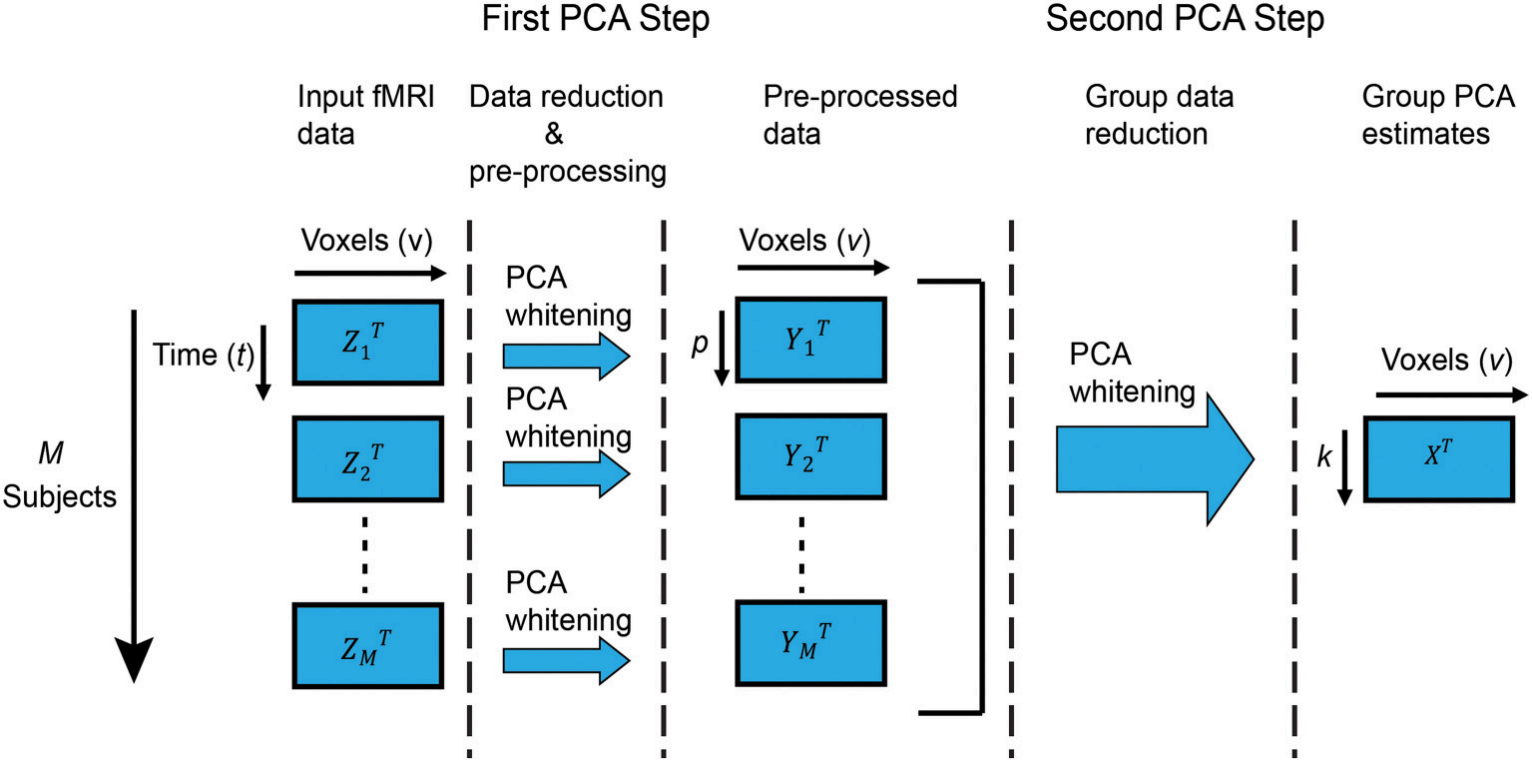
**Group PCA framework**. Graphical representation of the two-step PCA approach to group-level PCA we consider in this work. The first step performs subject-level PCA on each subject, followed by (optional) whitening. The second step performs group-level PCA on the stacked (concatenated) reduced-data from all subjects, and is the focus of our attention. Particularly, if time and memory resources were unlimited, standard PCA routines for subject-level PCA (such as EVD) would suffice for group-level PCA on the stacked data. For that reason, we consider that to be the “ideal” scenario and strive to replicate its results in the more realistic case of limited resources.

In this paper, we are interested in group ICA of fMRI data as originally described in Calhoun et al. (2001) and further expanded and reviewed in Erhardt et al. (2011) and Calhoun and Adali (2012). In this technique, *Z*_*i*_ are the fMRI data of subject *i* with dimension *v* × *t*, where *v* and *t* are the number of voxels and time points, respectively. *Z*_*i*_ is mean-centered on zero at each time point. Each subject's data is reduced along the time dimension using PCA to retain the top *p* components, which are then whitened^1^. Following, all *M* subjects are stacked along the (reduced) temporal dimension. Let *Y* = [*Y*_1_, *Y*_2_, …, *Y*_*M*_] be the temporally concatenated data where *Y*_*i*_ is the zero-mean *v* × *p* PCA-reduced data of subject *i*. Group-level PCA is then performed on the temporally reduced concatenated data, resulting in *k* group principal components in the group-level PCA space *X*. Figure 1 shows a graphical representation of group PCA.

Group ICA is regularly being used to analyze large numbers of subjects (Biswal et al., 2010; Allen et al., 2011), and multi-band EPI sequences (Feinberg et al., 2010; Setsompop et al., 2012) put even more memory demands on group PCA if the number of components retained in the first PCA step is increased significantly. We previously implemented some memory efficient ways to solve the PCA problem on large datasets in the GIFT^2^ toolbox using EVD, SVD and EM PCA. Options are provided in the GIFT toolbox to select the appropriate PCA method based on the problem size and computer RAM requirements. In this paper, we present ways to further accelerate the group-level PCA step using new algorithms, and discuss the scalability of PCA algorithms based on the problem size.

### Group-level PCA

Group ICA of temporally concatenated fMRI can be used to identify either spatial independent components (Calhoun et al., 2001; Allen et al., 2011) or temporal independent components (Smith et al., 2012). In spatial group ICA, subject-level PCA is typically carried out to reduce the time dimension prior to group-level PCA, as described in Figure 1. However, in temporal group ICA, subject-level PCA to reduce the spatial dimension is neither required nor recommended^3^; instead, group-level PCA is carried out directly on the input fMRI data *Z*_*i*_. In the following, we present algorithms for PCA estimation assuming the case depicted in Figure 1 and deferring any comments about temporal group-level PCA to the Discussion Section.

In the following, we present a selection of approaches for group PCA, starting with the traditional EVD method, which we consider the standard for accuracy in later comparisons. Then, based on considerations made on the EVD method and properties of the fMRI data, two approaches are proposed for efficient approximate estimation of the group PCA solution, namely subsampled voxel PCA (SVP) and subsampled time PCA (STP). Both approaches are useful for efficient initialization and accelerated convergence of the highly accurate randomized methods presented later. Following, Large PCA, a recent block Lanczos method with high accuracy and potential for application in large group PCA, is introduced for the purpose of comparison. Power methods, including the introduction of our novel MPOWIT technique, are discussed next. The connections and implications of MPOWIT on the popular expectation maximization PCA (EM PCA) approach are presented lastly. At every stage, we strive to present every algorithmic improvement and theoretical development in the context of fMRI data under various conditions. Nevertheless, all considerations should be straightforwardly extensible to other modalities and datatypes. Throughout the following, *F* is the eigenvector matrix required for GICA1 back-reconstruction (Erhardt et al., 2011). Finally, we assume that *k* < < *Mp* and *k* < < *v* in all time complexity assessments presented hereafter, unless otherwise noted.

#### Eigenvalue decomposition (EVD)

Using the EVD approach, the group-level PCA space *X* can be determined from the temporally stacked (concatenated) zero-mean data *Y* as follows:

- Compute the sample covariance matrix *C* in the smallest dimension of the data (Wang et al., 2006). If *Mp* < *v*, the *Mp* × *Mp* covariance matrix is:
  (1) $$\begin{array}{l} C=\frac{Y^{T}Y}{v-1}. \end{array}$$
- EVD factorizes the (symmetric) covariance matrix *C* into eigenvectors *F* and eigenvalues Λ:
  (2) $$\begin{array}{l} C=F\Lambda F^{T}. \end{array}$$
- The *v* × *k* group-level PCA space *X* is obtained by projecting the top *k* eigenvectors (columns) of *F* with largest eigenvalues onto the data, as shown below:
  (3) $$\begin{array}{l} X_{v\times k}=Y_{v\times Mp}F_{Mp\times k}\Lambda_{k\times k}^{-1∕2}. \end{array}$$

From Equation (1) and the description in Figure 1, we note that *C* has structure and could be visualized as a cross-covariance matrix between the *M* subject-level PCA components, with $C_{ij}=\frac{Y_{i}^{T}Y_{j}}{v-1}=C_{ji}$ as shown below:

(4) $$\begin{array}{l} C=\left[\begin{array}{cccc} C_{11} & C_{12} & \ldots & C_{1M}\\ C_{21} & C_{22} & \ldots & C_{2M}\\ \vdots & \vdots & \vdots & \vdots \\ C_{M1} & C_{M2} & \ldots & C_{MM} \end{array}\right]. \end{array}$$

Exploiting this structure, *C* can be computed with only two datasets in memory at a time, instead of stacking the entire data to form *Y*, leading to fewer computations and memory usage. However, as *M* increases, computation of the cross-covariance matrix becomes very slow since it requires $\frac{M\left(M-1\right)}{2}$ steps and an equal number of dataloads (hard disk swaps and data transfers). Therefore, EVD of *C* is a fast solution for small datasets but can quickly become inefficient on very large datasets. Moreover, Equation (3) incurs *M* additional dataloads, though it allows a memory efficient implementation with only one dataset in memory at a time. Also, note that computing the covariance matrix itself has time complexity *O*(*v*(*Mp*)^2^) when *Mp* < *v* and *O*(*Mpv*^2^) when *v* < *Mp*. The latter, however, has the convenience of requiring only one dataload per subject. Even better, in that case the covariance matrix in the voxel dimension, defined as $C^{v}=\frac{1}{v-1}YY^{T}$ in order to retain the same eigenvalues as *C*, can be written as a sum of subject-specific covariances $C_{i}^{v}$, and an efficient approach to compute the EVD of *C*^*v*^ is:

(5) $$\begin{array}{l} C^{v}=\overset{M}{\underset{i=1}{\sum }}C_{i}^{v}=\chi \Lambda \chi^{T}. \end{array}$$

The final estimate of the group-level PCA space is obtained as $X=\chi \sqrt{v-1}$. Equation (5) gives an upper bound on the *memory* required for group ICA of fMRI using the EVD method [i.e., *O*(*v*^2^) bytes], and note it only requires *M* dataloads. Particularly, for large-*M* region of interest- (ROI-) based group ICA *v* ≪ *Mp*. Thus, the left-hand equality in Equation (5) is highly efficient in memory for big ROI studies and should be the method of choice for computation of *C*^*v*^ rather than stacking the entire data in memory to form *Y*.

Clearly, trade-offs exist between time, memory, and dataloads depending on the exact values of *v*, *M*, and *p*. In some cases, which we consider later, it might be worth giving up on some computing speed in exchange for a largely disproportional improvement (reduction) in dataloads or memory footprint, and vice-versa, especially if numerical accuracy with respect to EVD of *C* is not compromised. This will be a recurring theme in the following sections.

#### Subsampled PCA

While EVD is very well-developed and accurate, it still becomes computationally and memory intensive when applied to large data^4^ because it requires computation of large covariance matrices. For that reason, before discussing other PCA methods, we introduce the concept of “subsampling” (i.e., partitioning the data into subsets) and propose its use to efficiently determine an approximate initial PCA space that can be incrementally refined in a multi-stage approach. We consider two methods for subsampling, both of which could be used to efficiently initialize the PCA subspace of the randomized PCA methods discussed later. In the first method, data is subsampled in voxel space whereas in the second method the time dimension is subsampled. While voxel and time are specific to fMRI data, the concept of subsampling can be easily extended to any other data modality.

##### Subsampled voxel PCA (SVP)

Subsampled voxel PCA (SVP) works by setting up a scenario in which *v* < *Mp* so that Equation (5) can be used to efficiently approximate *X* with only a few dataloads per subject. First, consider that, typically, fMRI data is spatially smoothed during the initial preprocessing to improve signal-to-noise ratio (SNR), which introduces data dependency in the spatial domain. Therefore, the actual number of independent and identically distributed (i.i.d.) samples present in the data is less than the voxel dimension *v* (Li et al., 2007). A set of approximately independent samples could be obtained by subsampling the data in the voxel dimension. In our experiments, we selected a value of 2 for subsampling depth in x, y, and z directions as this only reduces the number of in-brain voxels by a factor of 2^3^ = 8, i.e., *v*′ = *v*∕8. Thus, the resulting covariance matrix is still fairly approximate to the original one. All-odd and all-even subsampling depths are processed separately to estimate eigenvectors *F*_*a*_ and *F*_*b*_, respectively, using EVD with Equation (5) since typically *v*′ < < *Mp*. *F*_*a*_ and *F*_*b*_ are projected onto the data *Y* (Equation 8) in order to bring *X*_*a*_ and *X*_*b*_, respectively, back to dimension *v* from *v*′. These are finally stacked in the time dimension to determine a final (common) PCA subspace (Equations 9 and 10):

(6) $$\begin{array}{r} C_{aa}^{v^{\prime }}=X_{a}\Lambda_{a}X_{a}^{T},C_{bb}^{v^{\prime }}=X_{b}\Lambda_{b}X_{b}^{T}, \end{array}$$

(7) $$\begin{array}{r} F_{a}={\left(X_{a}^{T}Y_{a}\right)}^{T},F_{b}={\left(X_{b}^{T}Y_{b}\right)}^{T}, \end{array}$$

(8) $$\begin{array}{r} X_{a}=YF_{a},X_{b}=YF_{b}, \end{array}$$

(9) $$\begin{array}{r} \left[\begin{array}{cc} \frac{X_{a}^{T}X_{a}}{v-1} & \frac{X_{a}^{T}X_{b}}{v-1}\\ \frac{X_{b}^{T}X_{a}}{v-1} & \frac{X_{b}^{T}X_{b}}{v-1} \end{array}\right]=W\Lambda W^{T}, \end{array}$$

(10) $$\begin{array}{r} X=\left[X_{a},\;X_{b}\right]W\Lambda^{-1∕2}. \end{array}$$

*Y*_*a*_ and *Y*_*b*_ refer to subsampled data in odd- and even-voxel spaces, respectively. *F*_*a*_ and *F*_*b*_ are estimated in subsampled space *v*′ from covariance matrices $C_{aa}^{v^{\prime }}$ and $C_{bb}^{v^{\prime }}$, respectively, using Equations (6) and (7). A very high number of components (i.e., much larger than *k*; here, around 500, assuming *k* ≈ 100) are estimated in this intermediate PCA stage (Equation 6) to minimize error due to approximation. At the end of the estimation, only the *k* dominant components are extracted from *X* (Equation 10). Note that the use of Equation (5) for EVD of $C_{aa}^{v^{\prime }}$ and $C_{bb}^{v^{\prime }}$ allows SVP to operate in unstacked way (i.e., loading each subject's dataset at a time instead of stacking all datasets to form *Y*) and would require at most two dataloads per subject [one for Equation (6) and one for Equations (7) and (8)].

SVP is much faster compared to EVD as the voxel dimension is smaller by at least a factor of 8 but only gives an approximate PCA solution. SVP PCA estimates are a great initial solution for any of the randomized PCA methods discussed later, inducing to considerably faster convergence.

##### Sub-sampled time PCA (STP)

The time (stacked) dimension increases as more and more subjects are analyzed in a group PCA framework (Figure 1). By default, initial versions of the GIFT toolbox (Calhoun et al., 2004) used a three-step data reduction method for large dataset analysis in order to reduce the memory requirements from the group PCA framework of Figure 1. This three-step reduction operated as follows: (1) reduced datasets from the first PCA step (*Y*_*i*_) were randomly organized in groups of size *g* = 4; (2) PCA was applied on each group separately (including whitening^5^); (3) reduced group datasets were concatenated and a final PCA step was applied. This approach had the following shortcomings: (1) whitening in the intermediate group PCA (step two above) normalized the variance of components from each group and, therefore, the principal component weights were not correctly reflected in the final PCA step; (2) error of approximation increased if a low number of components was estimated in the intermediate group PCA step; (3) memory overhead increased if higher number of components were estimated in the intermediate group PCA step.

Here, we present a modified version of this three-step data reduction, which we call sub-sampled time PCA (STP). It estimates the PCA subspace *X* by incremental updates based on a different group (“sub-sample”) of subjects stacked in time. First, we do not use whitening in the intermediate group PCA (step two above). Second, the final group PCA space is incrementally updated, incorporating the estimates from the previous group PCA before the next group is considered. This reduces the memory overhead incurred by temporal concatenation. Third, a high number of components (around *k*′ = 500) is estimated in every group PCA update. The following equations summarize the proposed STP procedure for *Mp* < *v*:

(11) $$\begin{array}{r} C_{g}=\frac{Y_{g}^{T}Y_{g}}{v-1} \end{array}$$

(12) $$\begin{array}{r} C_{g}=F_{g}\Lambda_{g}F_{g}^{T} \end{array}$$

(13) $$\begin{array}{r} X_{g}=Y_{g}F_{g} \end{array}$$

(14) $$\begin{array}{r} \left[\begin{array}{cc} \frac{X_{g}^{T}X_{g}}{v-1} & \frac{X_{g}^{T}X_{g+1}}{v-1}\\ \frac{X_{g+1}^{T}X_{g}}{v-1} & \frac{X_{g+1}^{T}X_{g+1}}{v-1} \end{array}\right]=W\Lambda W^{T} \end{array}$$

(15) $$\begin{array}{r} X_{g}=\left[X_{g},X_{g+1}\right]W. \end{array}$$

*C*_*g*_ and *F*_*g*_ are the *gp* × *gp* covariance matrix in (stacked) time dimension and the eigenvectors of a group's data *Y*_*g*_, respectively. Assuming *gp* ≥ *k*′, eigenvectors *F*_*g*_ are projected onto the data *Y*_*g*_ to obtain a *v* × *k*′ subgroup PCA subspace *X*_*g*_ (Equation 13). Equations (11)–(13) are repeated for the next group to compute PCA estimates for data *Y*_*g*+1_. PCA estimates of group *Y*_*g*_ and group *Y*_*g*+1_ are stacked in time dimension and the common *v* × *k*′ PCA subspace *X*_*g*_ is obtained using Equations (14) and (15), since typically 2*k*′ < *v* in this case. Equations (11)–(15) are repeated until the last group is loaded. Only the *k* dominant PCA components are retained from the final matrix *X*_*g*_ at the end of the estimation: $X=X_{g}\Lambda^{-1∕2}$.

STP requires only a single pass through the data to determine an approximate PCA space and is a very useful method when data loading is a bottleneck. Both the estimation accuracy and memory requirements are proportional to the number of subjects included in each group and number of components estimated in the intermediate group PCA. In this paper, we select number of subjects in each group as *g* = 20, which has a small memory burden and yet gives a great approximation to the group PCA solution. Of note, STP not only generalizes the original three-step PCA approach but also includes MELODIC's incremental group PCA (MIGP; Smith et al., 2014) as a special case when *g* = 1.

#### Large PCA

Large PCA (Halko et al., 2011a) is a randomized version of the block Lanczos method (Kuczynski and Wozniakowski, 1992) that estimates a low rank PCA approximation of matrix *F* [see Equation (2)]. In block Lanczos methods, intermediate subspace estimates from every previous iteration are retained, each forming an additional “block” for the next iteration. This is different from subspace iteration (discussed next), which *updates* the PCA estimates instead, refining them until convergence is achieved. Similar to subspace iteration, Large PCA also exploits the powers of *YY*^*T*^ to obtain the reduced PCA space *X*. The Large PCA algorithm operates as follows:

A Krylov subspace *Kr* based on powers of the *YY*^*T*^ matrix is generated iteratively from an initial standard Gaussian random matrix *F*_0_ of size *Mp* × *b*, where *b* is the block length (typically, slightly larger than *k*):

(16) $$\begin{array}{l} Kr=\left[X_{0},X_{1},\ldots ,X_{j}\right], \end{array}$$

where *F*_0_ = *G*_*Mp* × *b*_, *X*_0_ = *YF*_0_, and $X_{j}=Y{\left(X_{j-1}^{T}Y\right)}^{T}=\overset{M}{\underset{i=1}{\sum }}Y_{i}{\left(X_{j-1}^{T}Y_{i}\right)}^{T}$.

*Kr* is of size *v* × (*j* + 1)*b*, where *v* is the number of voxels, and *j* ≥ 1 is the number of additional blocks required to obtain an accurate solution. The formation of *Kr* requires (*j* + 1)*M* dataloads and only one subject's dataset in memory at a time (unstacked *Y*). Of course, if enough RAM is available in the system to retain all subject's datasets in memory simultaneously (stacked *Y*), then *M* dataloads would suffice to compute *Kr* and also Equations (17)–(21) below.

After *Kr* is formed, an economy-size QR decomposition is performed on it (the columns of χ are orthonormal and *R* is an upper triangular real valued matrix):

(17) $$\begin{array}{l} Kr=\chi R. \end{array}$$

Following, χ is projected onto the data matrix as follows:

(18) $$\begin{array}{l} F=Y^{T}\chi , \end{array}$$

and compute an economy-size SVD on matrix F:

(19) $$\begin{array}{l} F=FSW. \end{array}$$

In order to obtain the PCA space *X*, the matrix product below is more efficient than Equation (3) because it does not require the additional dataload:

(20) $$\begin{array}{l} X=\chi W\sqrt{v-1}. \end{array}$$

Finally, retrieve only the first *k* dominant columns of *X* and *F*, and use the first *k* rows and *k* columns of *S*. Note that Equation (18) requires *M* additional dataloads, for a total of (*j* + 2)*M* dataloads in Large PCA with fixed *j* (unstacked *Y*).

The choice of *j* is problem dependent: for fixed dataset and datatype, increasing *j* dictates the attainable accuracy with respect to EVD; on the other hand, a fixed *j* gives different accuracy for different datasets and datatypes. Thus, the recommendation in the original publication (Halko et al., 2011a) was to set *b* to a large-enough value that would guarantee accuracy of the solution for a given data type, using fixed *j* = 2. While this approach guarantees a small number of dataloads, it does so by increasing the memory burden, due to larger *b*. In our experiments using the recommended settings, we have noticed that the memory usage^6^ incurred was much undesirable for large fMRI datasets. Moreover, the attained accuracy with respect to EVD seemed inconsistent across different fMRI datasets, suggesting every new dataset would require specific adjustments for better accuracy. Although increasing the size of *Kr* with larger *b* and/or larger *j* improves accuracy, without a direct check for convergence only blind adjustments are possible with the recommended approach. We then noticed that convergence could be assessed by computing the norm of the difference between the top *k* singular values of sequentially increasing *Kr* (Equation 16) and verifying that it meets some tolerance δ, as indicated in Equation (21). However, this implies that Equations (17)–(19) need to be computed for each *X*_*j*_ increment to *Kr*. Besides the additional computational burden, this increases the number of dataloads to (2*j* + 1)*M*. Based on an analysis presented in the Appendix of Supplementary Material (see Section Parameter Selection for Large PCA) a good compromise was to fix *b* = 170, generate the initial *Kr* with *j*_0_ = 6 before the first estimation of the singular values (Equation 21), and continue to augment *Kr* and estimate its singular values for *j* > *j*_0_ until convergence was attained. Our approach incurs a total of *j*2*M* − *j*_0_*M* + 2*M* dataloads for *j* > *j*_0_ and some additional computational effort, but controlled memory usage, all while guaranteeing the accuracy of the solution with respect to EVD. Finally, Equation (20) is computed only after convergence is attained.

(21) $$\left\|S_{j}-S_{j\;-\;1}\right\|<\;\delta .$$

The time complexity of large PCA over all *j* > *j*_0_ iterations is *O*(*Mpvbj*). Since both Equations (16) and (18) can be implemented with a “for loop,” only one subject's dataset is required in memory at a time. Thus, Large PCA (un-stacked) would be suitable for large group-level PCA of fMRI data. Finally, when Large PCA is initialized with STP or SVP, *X*_0_ is set to equal *X*^*STP*^ or *X*^*SVP*^, respectively, and we recommend *j*_0_ = 1 since convergence is attained much faster with this initialization.

#### Multi power iteration (MPOWIT)

Power iteration is an iterative technique which uses powers of the covariance matrix $C^{v}=\frac{1}{v-1}YY^{T}$ to estimate one component (a single column of *X*) at a time, with subsequent components determined after removing the variance associated with previous components from the data (known as deflationary mode). While the powers of *C*^*v*^ contain the same eigenvectors (*X*) as *C*^*v*^ itself, the largest eigenvalues become more dominant, emphasizing the direction of largest variability. However, power iteration techniques require a normalization step to avoid ill-conditioned situations. Different normalization approaches and the choice of initial PCA subspace mark the key differences among power iteration techniques. In traditional power iteration, the *L*_2_-norm of the PCA estimates is used for normalization in each iteration, as shown below:

(22) $$\begin{array}{r} x_{0}=G_{v\times 1}, \end{array}$$

(23) $$\begin{array}{r} x_{j}=C^{v}x_{j-1},j\geq 1, \end{array}$$

(24) $$x_{j}=x_{j}/{\left\|x_{j}\right\|}_{2}.$$

Subspace iteration (Rutishauser, 1970), also known as orthogonal iteration, extends power iteration to estimate multiple components simultaneously from the data (known as symmetric mode). It also uses powers of the covariance matrix $C^{v}=\frac{1}{v-1}YY^{T}$, iteratively estimating a subspace projection that contains the top *k* components of the PCA space *X*. It typically uses QR factorization, instead of *L*_2_-norm, to orthonormalize intermediate estimates *X*_*j*_ at each iteration and prevent them from becoming ill-conditioned. The following equations summarize subspace iteration (Saad, 2011):

(25) $$\begin{array}{r} X_{0}=G_{v\times k}, \end{array}$$

(26) $$\begin{array}{r} \chi_{j}=C^{v}X_{j-1},j\geq 1, \end{array}$$

(27) $$\begin{array}{r} X_{j}=orth\left(\chi_{j}\right), \end{array}$$

where *orth*(·) is an operation that returns an orthonormal basis, such as the QR factorization. The algorithm is initialized with a *v* × *k* Gaussian random matrix. *X*_*j*_ is the subspace estimate at the *j*^*th*^ iteration. Equations (26) and (27) are iterated until convergence. Subspace iteration is straightforward to implement but has slow convergence, especially for the last few eigenvalues, which converge much more slowly. Preconditioning techniques like shift-and-invert and Chebyshev polynomial (Saad, 2011) have been used on the covariance matrix to accelerate the subspace iteration method. Still, computing the covariance matrix is costly when the data are large. Hence, subspace iteration is not a popular method as compared to block Lanczos methods.

Here, we introduce a novel method called MPOWIT, which accelerates the subspace iteration method. It relies on making the projecting subspace larger than the desired eigen space in order to overcome the slow convergence associated with the subspace iteration approach. The MPOWIT algorithm starts with a standard Gaussian random matrix of size *v* × *lk*, following with an initial power iteration and the set of operations below:

(28) $$X_{0}=G_{v\times lk},\chi_{0}=\left(YY^{T}\right)X_{0},\Lambda_{0}=0,$$

(29) $$\begin{array}{r} X_{j}=orth\left(\chi_{j-1}\right)=\chi_{j-1}FL^{-1},j\geq 1, \end{array}$$

(30) $$\begin{array}{r} \chi_{j}=\left(YY^{T}\right)X_{j}=Y{\left(X_{j}^{T}Y\right)}^{T}=\overset{M}{\underset{i=1}{\sum }}Y_{i}{\left(X_{j}^{T}Y_{i}\right)}^{T}, \end{array}$$

(31) $$\begin{array}{r} \frac{X_{j}^{T}\chi_{j}}{v-1}=W_{j}\Lambda_{j}W_{j}^{T}. \end{array}$$

where *v* is the number of voxels, *l* is an integer multiplier, and *k* is the number of desired eigenvectors. The main innovation in MPOWIT stems from the realization, through experience, that a small fraction of the top principal components converges much faster than the rest. Thus, a larger subspace leads to fast retrieval of the top *k* components when *k* is only a fraction of that subspace's dimensionality. We also propose a faster implementation of *orth*(·) for MPOWIT to return an orthonormal basis for the column space of its operand efficiently. Since *lk* is typically small compared to the rank of the data, Equations (2) and (3) can be used as follows: first, perform a full EVD of $\chi_{j-1}^{T}\chi_{j-1}=FDF^{T}$ to obtain F, followed by $X_{j}=\chi_{j-1}FL^{-1}$ where *L* is a diagonal matrix containing the *L*_2_-norm of each column of χ_*j*−1_F. This strategy is typically two or three times faster than economy-size QR factorization (based on the default MATLAB implementations) and not memory intensive for *lk* ≤ 500 (for *k* = 100, we set *l* = 5 based on the analysis in Appendix Section How to Select the Projecting Subspace Size (*l*) for MPOWIT?; Supplementary Material). Furthermore, since computing *YY*^*T*^ on large data is inefficient in memory, the associative matrix multiplications shown in the center and right hand side of Equation (30) are used instead. Finally, Equation (31) is the EVD of $X_{j}^{T}Y{\left(X_{j}^{T}Y\right)}^{T}$, which is implemented using the function *eigs*(·)^7^ in MATLAB to efficiently retrieve only the top *k* eigenvalues Λ_*j*_. Equations (29)–(31) are iterated for *j* ≥ 1 until the top *k* eigenvalues in the subspace projection converge to within the specified tolerance δ, as shown in Equation (32), and the choice of Λ_0_ = 0, where 0 is a *k* × *k* matrix of zeros, guarantees the algorithm will not stop before *j* = 2:

(32) $$\left\|\Lambda_{j}-\Lambda_{j\;-\;1}\right\|<\delta .$$

After convergence, the reduced PCA space *X* is obtained as:

(33) $$\begin{array}{r} X=X_{j}W_{j}\Lambda_{j}^{-1∕2}, \end{array}$$

and the eigenvectors *F* follow from *F* = *Y*^*T*^*X*, then normalize the columns of *F* to unit *L*_2_-norm. A rank *k* PCA approximation is obtained by retrieving the first *k* dominant columns of the matrices *X* and *F*.

The time complexity of MPOWIT over all *j* iterations is *O*(*Mpvlkj*). Based on Equation (30), the algorithm requires only *M* dataloads per iteration and a total of (*j* + 1)*M* dataloads, which makes MPOWIT scalable for large data analysis. Furthermore, based on the original power iteration algorithm, simple *L*_2_-norm normalization of the columns of *X*_*j*_ without full orthogonalization would suffice in Equation (29). However, in our experiments, we determined that assessments about the convergence of the algorithm are considerably more reliable if they are based on the eigenvalues Λ_*j*_ obtained from orthogonal *X*_*j*_ instead. Thus, explicit orthonormalization of *X*_*j*_ in each iteration is preferred.

Also, when MPOWIT is initialized with STP or SVP, *X*_0_ is set as the top *lk* components of *X*^*STP*^ or *X*^*SVP*^, respectively, and $\Lambda_{0}=\Lambda^{STP}$ or $\Lambda_{0}=\Lambda^{SVP}$ since all eigenvalues are available at the end of the STP and SVP procedures. Initializing with a sub-sampling technique accelerates convergence and, more importantly, prevents dataloads from becoming a bottleneck in the analysis pipeline. Lastly, note that MPOWIT differs from a block version of subspace iteration (Mitliagkas et al., 2013) where regular subspace iteration is applied in “online” or “streaming memory” mode, i.e., making a single pass over the data. Although, this approach minimizes the number of dataloads (to *M*), the PCA solution is only approximate with respect to the EVD solution and, thus, not recommended for group ICA of fMRI data.

#### MPOWIT and expectation maximization PCA (EM PCA)

To complete our discussion on methods for PCA of large datasets, we present expectation maximization PCA (EM PCA). Our focus is on the connections and the implications that certain MPOWIT concepts have on this popular technique. EM PCA (Roweis, 1997) uses expectation and maximization steps to determine the PCA subspace. The algorithm operates as follows:

(34) $$\begin{array}{r} X_{0}=G_{v\times k}, \end{array}$$

(35) $$\begin{array}{r} F_{j}^{T}={\left(X_{j-1}^{T}X_{j-1}\right)}^{-1}X_{j-1}^{T}Y,j\geq 1, \end{array}$$

(36) $$\begin{array}{r} X_{j}=YF_{j}{\left(F_{j}^{T}F_{j}\right)}^{-1}. \end{array}$$

In Equation (34), a Gaussian random matrix of dimensions *v* × *k* is selected as the initial PCA subspace. In the expectation step (Equation 35), the PCA subspace *X*_*j*−1_ is fixed and the transformation matrix *F*_*j*_ is determined, while in the maximization step (Equation 36), *F*_*j*_ is fixed and the subspace *X*_*j*_ is determined. Equations (35) and (36) are iterated until the algorithm converges to within the specified error for tolerance as shown below:

(37) $$\left\|X_{j}-X_{j\;-\;1}\right\|<\delta .$$

After convergence, the reduced PCA space *X* is determined using Equations (31) and (33): first, perform a full EVD of $\frac{X_{j}^{T}{YY^{T}X}_{j}}{v-1}$, followed by $X=X_{j}W_{j}\Lambda_{j}^{-1∕2}$.

The time complexity of EM PCA over all iterations is only *O*(*Mpvkj*) but it takes a considerably larger number of iterations to converge when compared to Large PCA and MPOWIT methods. This is because EM PCA has the same convergence properties of subspace iteration. In fact, as we prove in Appendix Section Proof: MPOWIT and EM-PCA Converge to the Same PCA Subspace (*X*) (Supplementary Material), EM PCA returns the same subspace estimate as MPOWIT if both run for the same number of iterations and use the same initial guess. Naturally, the acceleration schemes used for subspace iteration [see Section Multi Power Iteration (MPOWIT) and Appendix Section How to Select the Projecting Subspace Size (*l*) for MPOWIT?; Supplementary Material] are equally applicable and useful for EM PCA. However, as seen from Equations (35) and (36), EM PCA requires loading the data into memory *twice* if un-stacked PCA is performed. Since dataloading is a bottleneck for very large group analyses, EM PCA is still slower than MPOWIT when PCA is carried out on un-stacked data (i.e., on each *Y*_*i*_ at a time rather than the entire stacked *Y* at once). Therefore, we forgo further comparisons with EM PCA in the Results Section.

As a final remark on methods, our MPOWIT method relates to normalized power iteration (Martinsson et al., 2010; Halko et al., 2011b). However, normalized power iteration is more a variation of the EM PCA method where both the expectation (Equation 35) and maximization (Equation 36) steps are orthonormalized. Hence, the subspaces in each iteration are the same for both EM PCA and normalized power iteration. Also, we note that orthonormalization of the expectation step (Equation 35) in normalized power iteration is redundant and, therefore, normalized power iteration has the same shortcomings as EM PCA in large un-stacked group analyses, i.e., both require two dataloads per iteration. Thus, the time required to solve group PCA is significantly longer than with MPOWIT when a large number of subjects are included [see definition of “large” in Section Sub-Sampled Time PCA (STP)].

### Data and preprocessing

We use 1600 pre-processed subjects from resting state fMRI data (a superset of the data presented in Allen et al., 2011) and perform group PCA. Pre-processing steps include image realignment using INRIalign (Freire et al., 2002), slice-timing correction using the middle slice as reference, spatial normalization (Friston et al., 1995) and 3D Gaussian smoothing with a kernel size of 10 × 10 × 10 mm. No normalization is done on the BOLD fMRI timeseries (e.g., dividing by the variance or mean as is sometimes done for ICA approaches). Scans from 3 to 150 are included in the analysis to match the same time-points across subjects. A common mask is applied on all subjects to include only in-brain voxels. The common mask for all the subjects is generated by using element-wise multiplication on the individual subject masks. A widely used approach to generate individual subject masks is to eliminate non-brain voxels by keeping voxels with values above or equal to the mean over an entire volume for each timepoint.

In the initial subject-level PCA step of group ICA (Calhoun et al., 2001), each individual subject's fMRI data of dimensions *v* × *t* is reduced to a few whitened principal components of dimensions *v* × *p* (see Figure 1). We use EVD to reduce subject specific fMRI data and retain *p* = 100 components, capturing near-maximal individual subject variability during the first PCA step (Erhardt et al., 2011). In the second PCA step (group-level PCA), data from each subject in the first PCA step is stacked along the (reduced) time dimension. As the number of subjects increases, the memory requirement increases since the data is temporally concatenated. On all reported experiments, without loss of generality, we used a typical fMRI acquisition setting where the whole scanning field is sampled at 3 × 3 × 3 mm voxel resolution (resulting in a matrix of dimensions 53 × 63 × 46) and the number of time points is 148 with TR = 2000 ms.

### Experiments

A number of experiments have been conducted to assess memory usage and computation time for all group PCA methods discussed previously. Firstly, we assessed memory usage for varying number of subjects (*M*). Next, for each group-level PCA algorithm tested in this paper, we conducted 20 different group ICA analyses, varying the number of subjects (*M*) used in each analysis by 100, 200, 400, 800, and 1600, and the number of components (*k*) by 25, 50, 75, and 100. The group-level PCA algorithms considered were EVD, Large PCA, multi power iteration (MPOWIT), subsampled voxel PCA (SVP), and subsampled time PCA (STP). For STP, the number of subjects in each group (*g*) was selected to be as large as possible and such that the analysis did not exceed 4 GB RAM (*g* = 20 in our analyses). Since, Large PCA and MPOWIT could also be carried out by loading one dataset at a time per PCA iteration, we also included the un-stacked group PCA cases in the analyses. In total, 7 × (5 × 4) = 7 × 20 = 140 different group-level PCA cases were considered for comparison in terms of their accuracy and required computing time. In addition, the number of iterations until convergence was assessed for Large PCA and MPOWIT on each scenario. Finally, we illustrated the total group ICA pipeline computing times attainable using the MPOWIT method (stacked and un-stacked) in the group-level PCA stage (including dataloading times).

## Results

### Memory requirements

**Figure 2:**
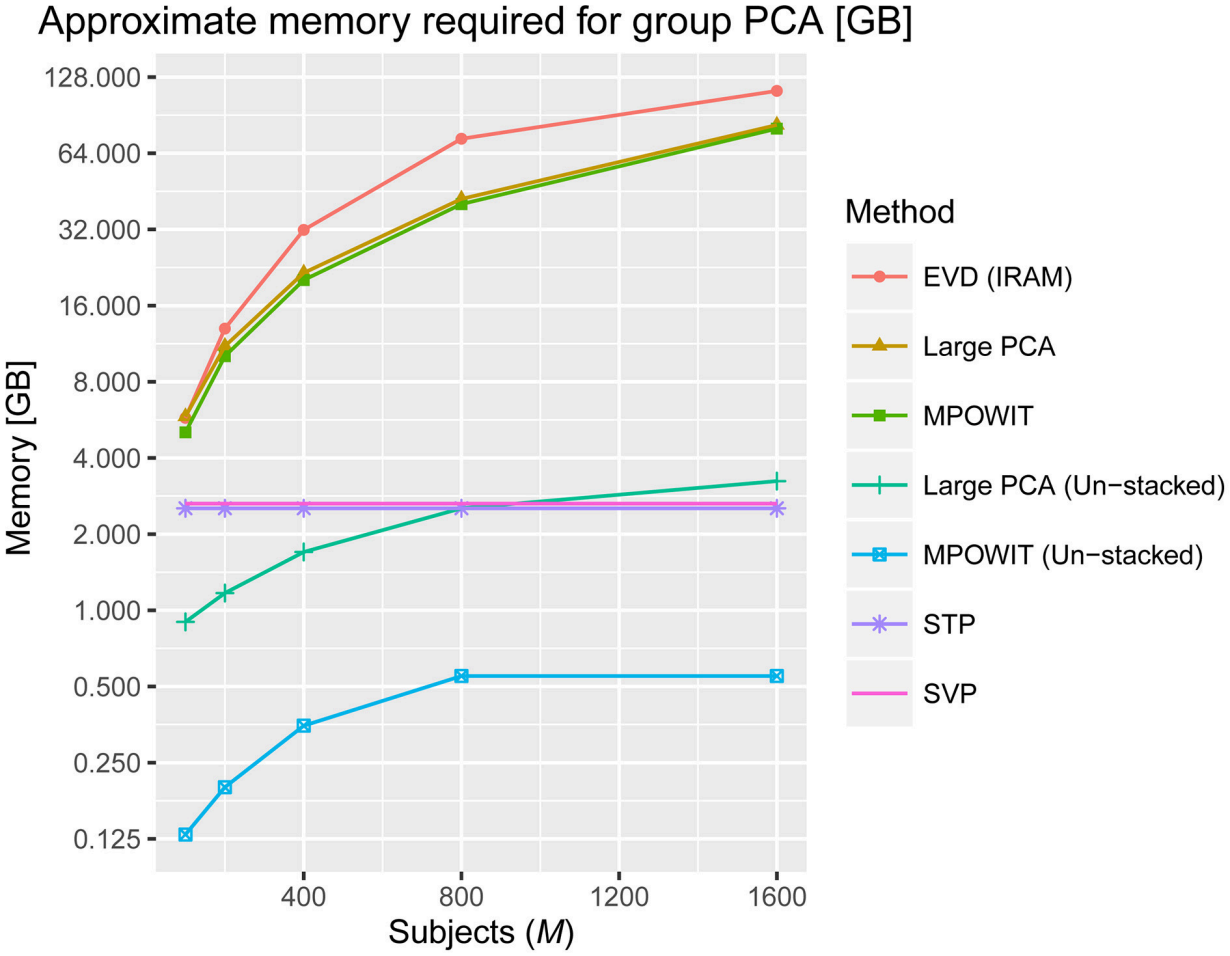
**Approximate memory required in gigabytes (GB) to solve the group PCA problem**. The number of in-brain voxels is *v* = 66745, the number of PCA components in the first PCA step is *p* = 100, the number of components in the second PCA step is *k* = 100 and the number of subjects (*M*) selected are 100, 200, 400, 800, and 1600. We used the number of block iterations *j* = 6 and block size *b* = 170 for Large PCA (see Appendix Section Parameter Selection for Large PCA; Supplementary Material), and the block multiplier *l* was set to 5 for MPOWIT (see Appendix Section How to Select the Projecting Subspace Size (*l*) for MPOWIT?; Supplementary Material). The number of subjects in each group (*g*) is set to 20 when estimating PCA using STP. We give the equations used to estimate the memory required by each PCA algorithm discussed in this paper in Appendix Section How Much Memory is Required during the Group-Level PCA Step? (Supplementary Material).

After applying a common binary mask to all time points, there were 66,745 in-brain voxels per time point. Figure 2 summarizes the memory requirements for each group PCA algorithm applied on this data, using the parameters specified in Section Accuracy, Computing Time, and Convergence. Note that for *M* ≥ 800, the voxel dimension is smaller than the stacked time dimension (i.e., *v* < *Mp*). Still, we cannot use Equation (5) to compute the covariance matrix in the voxel dimension because the voxel dimension is also very large and it may take several hours to compute if loading each dataset at a time. Thus, EVD is not scalable for large data analysis as both the memory burden to compute the covariance matrix and the computational burden to solve the eigenvalue problem increase exponentially. On the other hand, the un-stacked versions of randomized PCA approaches like Large PCA and MPOWIT are scalable for large datasets, meaning that Large PCA and MPOWIT could load each subject's dataset *Y*_*i*_ at a time for each PCA iteration. Thus, un-stacked versions of these algorithms are also considered. Subsampled EVD methods like SVP and STP are also considered as these have fixed memory requirements and are independent of the number of subjects analyzed.

Some notes about multi-band EPI sequences and subject-level PCA are in order here. First, even if the fMRI data were acquired at a 2 × 2 × 2 mm voxel resolution (roughly, *v* = 180000 in-brain voxels) and collected for 30 min using multi-band EPI sequences with TR = 200 ms, there would be no significant impact on the memory required to solve the first PCA step, including computations of the subject-specific covariance matrix. This is because the time dimension would still be considered “small” since *t* = 30 × 60 × 5 = 9000 < 10000. With less than 10,000 time points, the first PCA step could be easily solved by loading the data in blocks along the voxel dimension, summing covariance matrices of dimension *t* × *t* across blocks, i.e., $\overset{blocks}{\underset{n=1}{\sum }}{\left(C_{tt}\right)}_{n}=F\Lambda F^{T}$, and using the EVD (IRAM) method [*eigs*(·) function in MATLAB]. The memory required by EVD to solve the ensuing group-level PCA, however, increases with the number of components *p* retained in the first PCA step, the number of subjects *M*, and the number of in-brain voxels *v*, assuming the entire temporally stacked data *Y* is loaded in memory, which makes it unscalable. Unlike EVD, MPOWIT (un-stacked) would solve this group PCA problem using less than 4 GB RAM even if *p* was increased from 100 to 200 components and *v* was increased from 60000 to 180000 in-brain voxels, assuming *M* = 5000 subjects, highlighting its scalability strengths.

### Accuracy, computing time, and convergence

Here, we present results for the group PCA experiments described in Section Data and Preprocessing. If not specified otherwise, all processes were tested on a server running Linux Centos OS release 6.4 with 512 GB RAM, and MATLAB R2012a. We also note that the files with the results from the initial subject-level PCA step were always saved as uncompressed “.mat” files to later speed up the data loading process during the group PCA step. The parameter settings used to solve the group PCA problem for each algorithm are described below:

- **EVD**: The covariance matrix is always computed in the smallest dimension of the temporally stacked data. The IRAM algorithm is used to find the desired eigenvectors. In this study, we used MATLAB's *eigs* (·) function, which is built on the IRAM method. The maximum error tolerance selected was δ = 10^−6^ and the maximum number of iterations was set to 1000.
- **Large PCA**: Here, we used settings based on the Pareto-optimal study in Appendix Section Parameter Selection for Large PCA (Supplementary Material). The block length was set to *b* = 170, the number of initial block iterations *j*_0_ was set to 6 and the error tolerance was set to 10^−6^. Note that as the number of block iterations (*j*) executed until convergence increases, the performance of the algorithm decreases (i.e., higher memory and lower speed).
- **MPOWIT**: The maximum error tolerance and maximum number of iterations were set to 10^−6^ and 1000, respectively. The multiplier *l* was set to 5 (see Appendix Section How to Select the Projecting Subspace Size (*l*) for MPOWIT?; Supplementary Material) to improve the rate of convergence. As the value of *l* increases, the rate of convergence and accuracy increase but the computational performance decreases (i.e., higher memory and lower speed in each iteration).
- **SVP**: A value of 2 is selected as subsampling depth and 500 intermediate PCA components are estimated for odd and even voxel spaces.
- **STP**: The number of subjects included in each group is 20, and 500 intermediate PCA components are estimated per group.

The *L*_2_-norm of the absolute difference between the top *k* eigenvalues obtained from the randomized PCA methods and those from the EVD (IRAM) method were used to determine the accuracy of the estimated PCA components. The eigenvalues from the EVD method are always considered the ground-truth.

**Figure 3:**
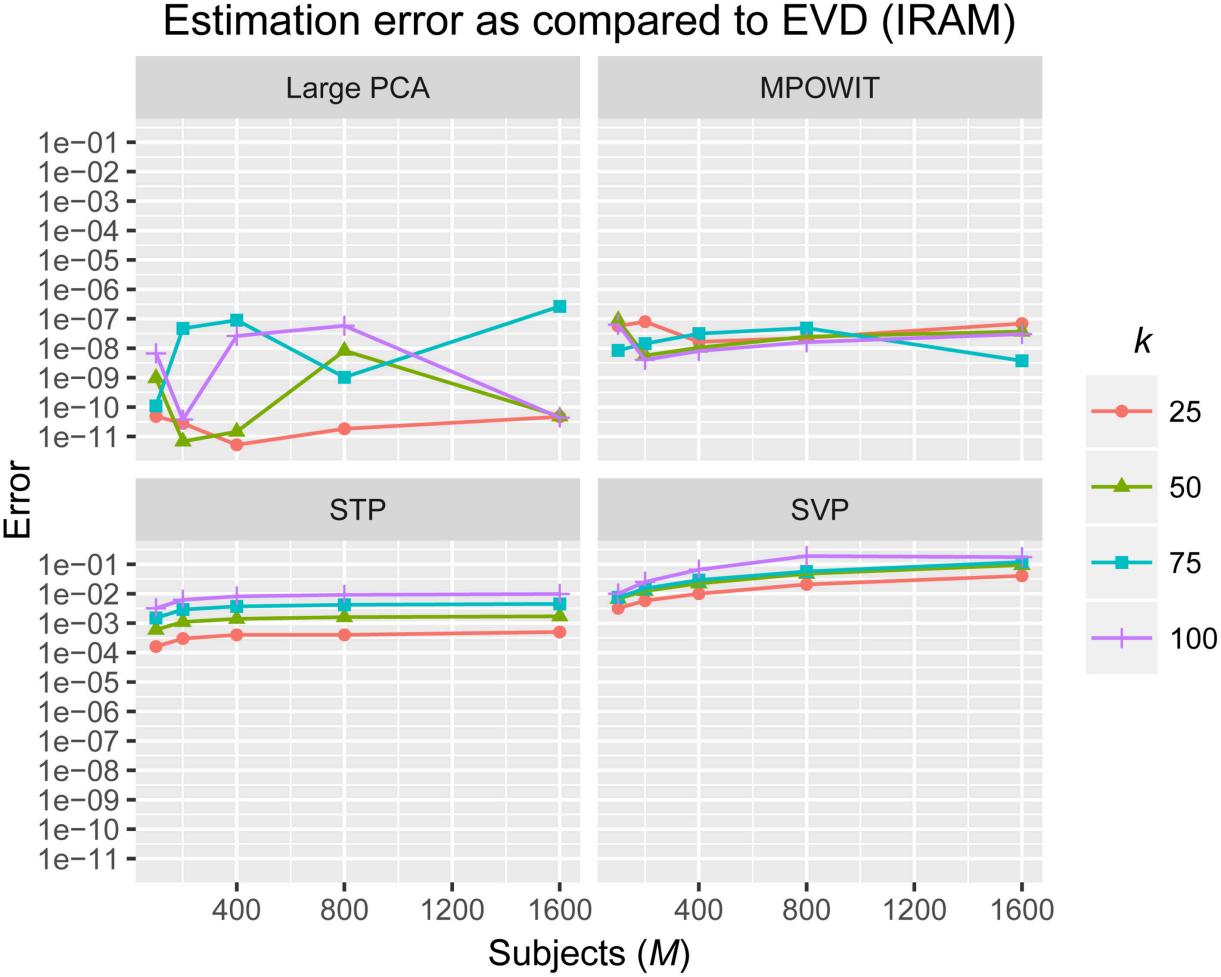
**Estimation error as compared to EVD (IRAM)**. *L*_2_-norm of error is computed between the eigenvalues of each method and the eigenvalues of the EVD method.

**Figure 4:**
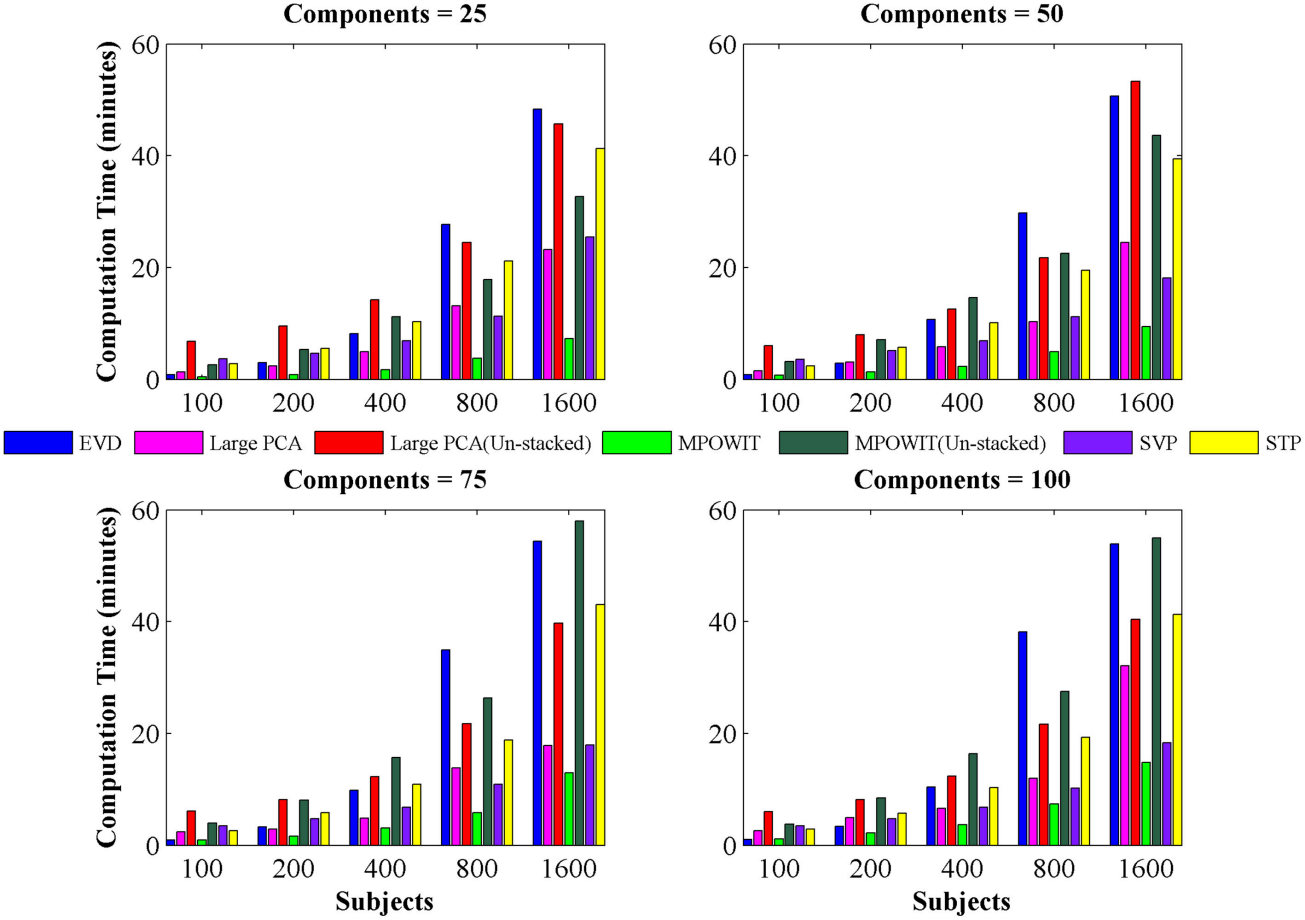
**Computing time (in minutes) taken to solve group-level PCA using EVD (IRAM), Large PCA, MPOWIT, SVP, and STP algorithms**. Using different numbers of subjects and components. The computing time of both Large PCA (un-stacked) and MPOWIT (un-stacked) are also reported.

**Figure 5:**
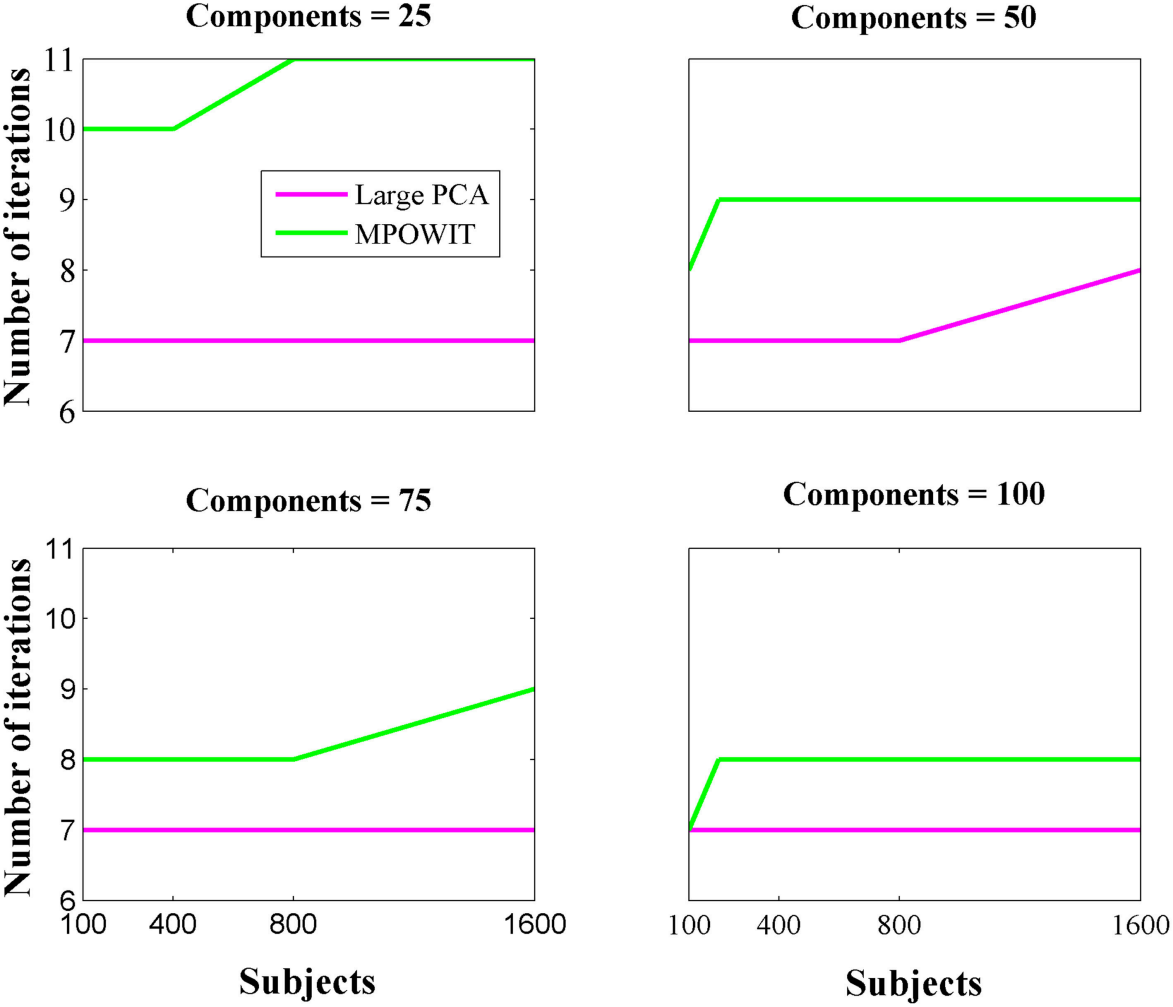
**Number of iterations required for convergence for both Large PCA and MPOWIT algorithms**.

From Figure 3, it is evident that both the Large PCA and MPOWIT methods give more accurate results than subsampled PCA methods SVP and STP across all model orders. Overall, STP estimates components with higher accuracy than SVP across all model orders. We also note that, generally, Large PCA estimates PCA components with slightly better accuracy than MPOWIT. Figure 4 compares the computing time taken in minutes by each algorithm to solve the group-level PCA problem. Subsampled PCA methods like SVP and STP outperform EVD and unstacked versions of MPOWIT and Large PCA at the cost of accuracy. MPOWIT and Large PCA outperform EVD when the entire data is loaded in memory, i.e., when *Y* fits in RAM. When the data is not loaded in memory, MPOWIT (un-stacked) marginally outperforms EVD and Large PCA (un-stacked) at lower model orders (*k* = 25 and *k* = 50) whereas at higher model orders (*k* = 75 and *k* = 100), Large PCA (un-stacked) marginally outperforms both EVD and MPOWIT (un-stacked). Finally, Figure 5 shows the number of iterations MPOWIT and Large PCA take to converge. Overall, Large PCA takes fewer iterations to converge than MPOWIT due to larger block sizes. However, note from Figure 2 that MPOWIT (un-stacked) requires considerably less memory than Large PCA (un-stacked) with fairly sublinear increase in memory use as *M* increases.

### Group ICA and subject back-reconstruction

**Figure 6:**
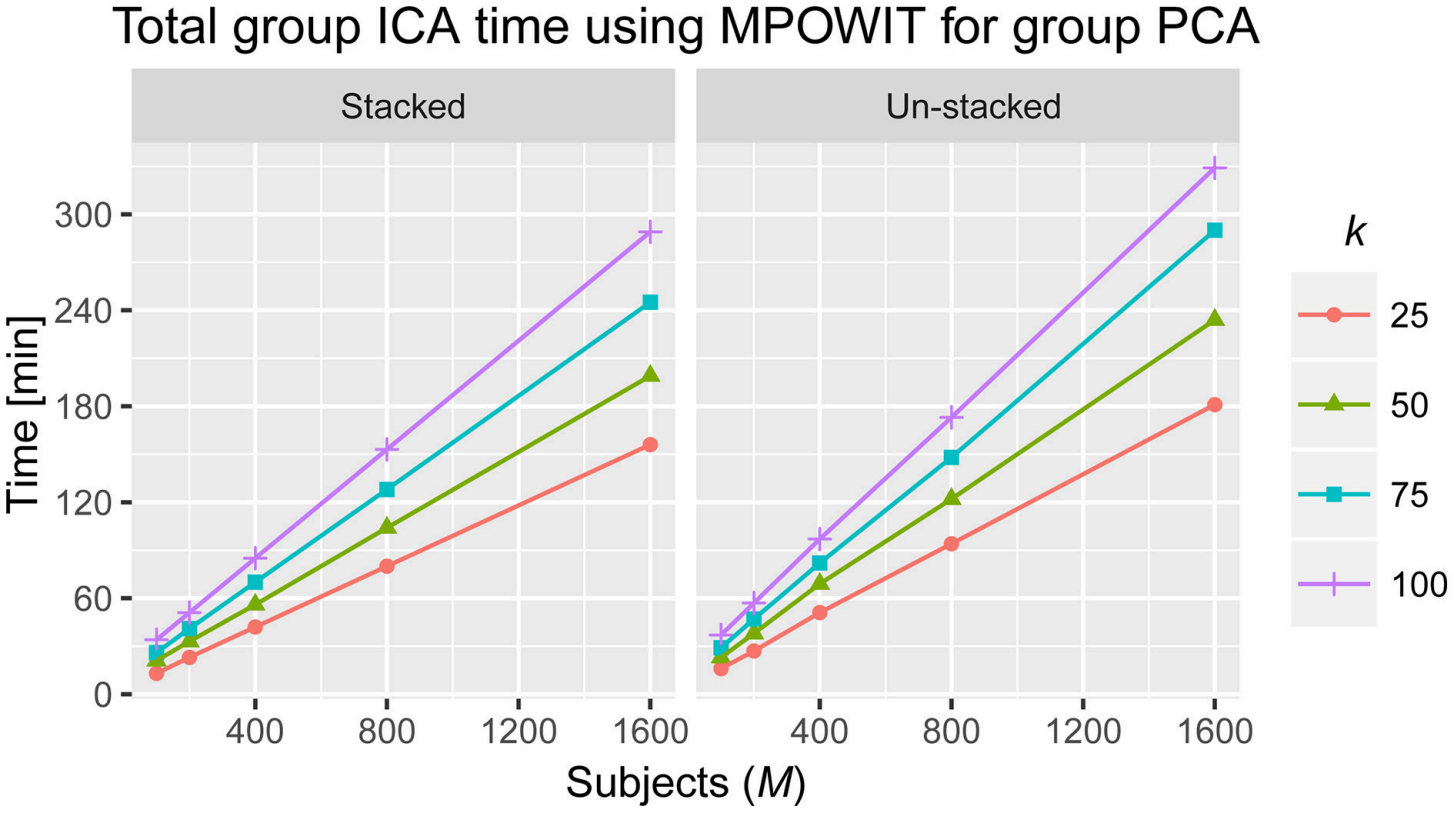
**Total time to solve the entire group ICA analysis using MPOWIT for the group PCA step (in minutes)**. Computing times when all the data is loaded in memory (stacked) and when datasets are loaded each at a time in every iteration (un-stacked).

Spatial ICA was performed on the final group-level PCA components to determine maximally statistically independent components. The Infomax ICA algorithm (Bell and Sejnowski, 1995) was repeated 10 times in an ICASSO framework (Himberg et al., 2004) with a different random initialization at each run. The most stable run estimates were used instead of using centrotype estimates (Ma et al., 2011). We used the GICA1 back-reconstruction method (Erhardt et al., 2011) to reconstruct individual subject component maps and timecourses for each analysis. Individual subject component maps and timecourses were then scaled to Z-scores. In Figure 6, we illustrate the total group ICA analysis computing times attainable using the MPOWIT method (stacked and un-stacked, respectively) in the group-level PCA stage (including dataloading time).

## Discussion

We demonstrated the entire group ICA process including the group PCA step on a Linux server with 512 GB RAM. We infer from Figure 2 that a large group ICA analysis using un-stacked versions of MPOWIT and Large PCA can be performed on machines with only 4 GB RAM. Moreover, the un-stacked versions of MPOWIT and Large PCA are designed to solve the group PCA problem by loading one dataset at a time. This is a nice feature which makes these algorithms ideal for PCA of “big data” where chunks of data can be extracted one at a time using memory mapping techniques.

Comparing among EVD, Large PCA, and MPOWIT, we notice that MPOWIT and Large PCA outperform EVD (IRAM) in terms of speed when all datasets are already loaded in memory. As depicted in Figure 4, pink (Large PCA) and light green (MPOWIT) bars are always shorter than the blue (EVD) bar. When only one subject's dataset can be loaded in memory at a time, in which case un-stacked MPOWIT and un-stacked Large PCA have to be used, the computation time increases as showed by the dark green (MPOWIT un-stacked) and red bars (Large PCA un-stacked) in Figure 4. Overall, blue and dark green bars are comparable at various model orders. Dark green bars marginally outperform dark red and blue bars at lower model orders whereas dark red bars marginally outperform dark green and blue bars at higher model orders. We note that un-stacked Large PCA uses larger block size (Krylov subspace) than un-stacked MPOWIT and, therefore, converges faster. In our experiments, both Large PCA and MPOWIT take at least seven iterations to converge to the PCA solution. With increasing datasets, data loading could be a big bottleneck in computational performance. To speed up the process further, we recommend using PCA estimates from subsampled PCA [Section Sub-sampled Time PCA (STP)] instead of random initialization. In our examples, MPOWIT and Large PCA methods provide very similar principal components, differing by less than 10^−6^.

The PCA methods we discussed in this paper are generic and can be applied to any dataset without any major modifications. However, our goal here is to demonstrate the applicability of these algorithms to real-valued fMRI data in the context of group ICA.

**Figure 7:**
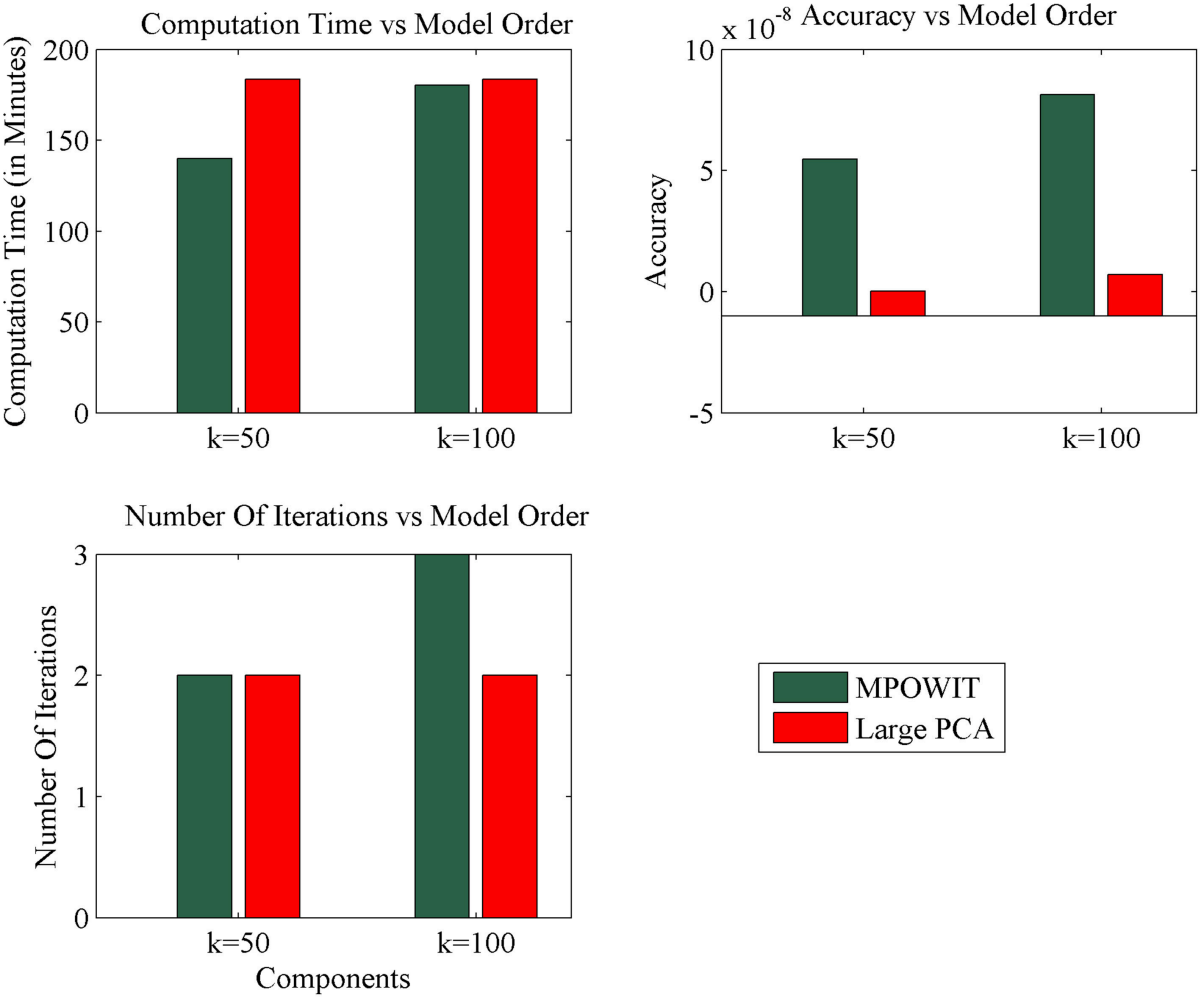
**Comparison of MPOWIT and Large PCA on Windows desktop with 4 GB RAM**. Initial PCA subspace is selected from STP method to accelerate algorithms MPOWIT and Large PCA for very large data (both un-stacked). Error in top row second column is computed using *L*_2_ - norm of difference between the eigenvalues obtained from the selected PCA method and EVD method.

The execution time for the largest group ICA analysis in this paper, i.e., 1600 subjects and 100 independent components, using the un-stacked version of our MPOWIT PCA algorithm for the group-level PCA, was 329 min (5.48 h, Figure 6). Reloading the datasets from the first PCA step was fast in our Linux server due to the Operating System's “cache effect.” We repeated the 1600 subject group-PCA problem with model orders 50 and 100 on a Windows desktop with 4 GB RAM using both MPOWIT and Large PCA. To speed up the PCA estimation of MPOWIT and Large PCA, 500 PCA components from subsampled PCA [Section Sub-Sampled Time PCA (STP)] were used as the initial PCA subspace. Since components estimated by STP method are more accurate than SVP method (Figure 3), STP components are used as initial subspace. Figure 7 shows that both MPOWIT and Large PCA successfully recovered 100 group PCA components with high accuracy in little more than 3 h (including the STP PCA estimation). MPOWIT required three iterations and large PCA required two block iterations to solve the largest group PCA problem in this paper which would have been an impossible problem to solve using EVD or assuming the entire (reduced) data from the first PCA step had to be loaded in memory during the group PCA step (Figure 2). A significant speedup can still be achieved if the entire group ICA process is run in parallel using, for example, GPU acceleration or distributed clusters.

Our results also emphasize that EVD is not preferred for large scale analysis as there is an extra cost in storing the covariance matrix in memory. One way to overcome this issue is based on Equation (5). If one dimension of the data is fixed, the covariance matrix can be computed in that dimension and the net covariance matrix could be computed by adding the covariance matrices across subjects. Moreover, since the covariance matrix is symmetric, only the lower or upper triangular portions need to be stored in memory (for eigenvalue problems). LAPACK^8^ eigen solvers such as DSPEVX or SSPEVX leverage this property and use only lower or upper triangular portions of the matrix. However, the performance (computing speed) of these algorithms will still be poor if the covariance matrix is very large.

The PCA methods applied for performing spatial group ICA are equally valid for temporal group ICA (Smith et al., 2012). In temporal group ICA, subject-level PCA is not performed and, thus, group PCA is computed directly on the preprocessed fMRI datasets stacked along the time dimension (Boubela et al., 2013). The memory requirements for the temporal group PCA step using the PCA methods we presented here could be computed using *p* = *t* (See Appendix Section How Much Memory is Required during the Group-Level PCA Step?; Supplementary Material). Notice that loading the full preprocessed fMRI datasets instead of loading just the subject-level PCA results (as in spatial group PCA) is considerably more time-consuming. Conveniently, our MPOWIT PCA approach supports processing un-stacked datasets and, thus, is highly suitable for very large temporal group PCA of multi-band EPI fMRI.

**Figure 8:**
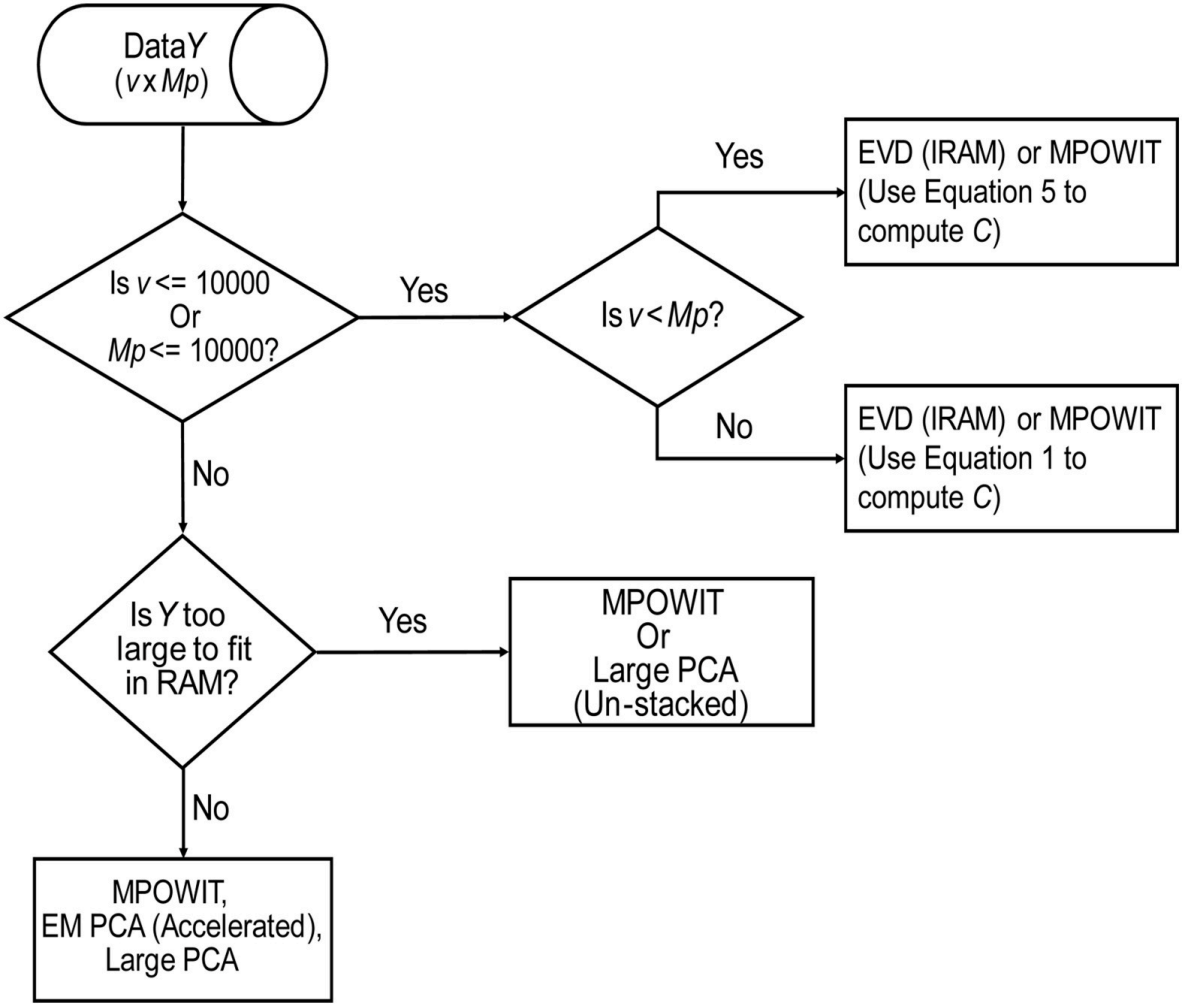
**PCA Algorithm selection**. This flowchart summarizes our recommendations for selection of the most appropriate PCA method given the dimensions of the data (v × Mp), where v is the number of in-brain voxels, *p* is the number of components retained in the first PCA step, and *M* is the number of subjects.

Based on the methods discussed here, we present our findings in a flowchart (Figure 8), indicating our recommendations for selection of the PCA method given the problem size and its order of complexity (*k*). The EVD (IRAM) method is preferred when the problem size is small (i.e., *v* ≤ 10000 or *Mp* ≤ 10000). Note, however, that MPOWIT could also be applied directly on the covariance matrix [Equation (30), left-hand side]. Thus, if the smallest dimension of the data is less than or equal to 10000, applying MPOWIT on the covariance matrix requires only a single dataload per subject and is more computationally efficient than Equation (30) (right-hand side), which requires one dataload per subject in each iteration. Finally, randomized PCA methods such as MPOWIT, EM PCA (accelerated), and Large PCA are efficient in memory and/or speed on large data problems that could be fit in the computer RAM. However, when the data is too large to fit in the computer RAM, un-stacked versions of MPOWIT or Large PCA are preferred with PCA subspace initialization from STP method. At most 2–3 iterations are required to estimate PCA components with high accuracy when the PCA subspace is initialized using STP. However, the performance of Large PCA is highly dependent on the correct tuning of *j*_0_ and *b* (see Appendix Section Parameter Selection for Large PCA; Supplementary Material) for each given dataset. MPOWIT, on the other hand, seems to produce comparable results with little need for tuning of *l* (see Appendix Section How to Select the Projecting Subspace Size (*l*) for MPOWIT?; Supplementary Material).

## Conclusions

We presented a new approach for PCA-based data reduction for group ICA called MPOWIT and demonstrated that it can efficiently solve the large scale PCA problem without compromising accuracy. The un-stacked version of MPOWIT takes almost the same time to complete the analysis as compared to EVD but requires much less RAM. We showed that MPOWIT enables group ICA on very large cohorts using standard fMRI acquisition parameters within 4 GB RAM. Computationally efficient data reduction approaches like MPOWIT are becoming more important due to the larger datasets resulting from new studies using high-frequency multi-band EPI sequences and from an increased tendency to share data in the neuroimaging community. Even in such challenging scenarios, un-stacked MPOWIT could realistically solve group-level PCA on virtually any number of subjects, limited only by time, not memory, constraints. Given its high scalability and right fit for parallelism, MPOWIT sets the stage for future groundbreaking developments toward extremely efficient PCA of big data using GPU acceleration and distributed implementations.

## Author contributions

SR—Developed PCA algorithm (MPOWIT) for data reduction. RS—Worked extensively on revising paper and helped with mathematical proofs. JL—Gave good feedback on improving the manuscript. VC—Gave good feedback on improving the manuscript and also work was done under his supervision.

## Funding

This work was funded by NIH 2R01EB000840, R01EB020407, and COBRE 5P20RR021938/P20GM103472 (Calhoun).

### Conflict of interest statement

The authors declare that the research was conducted in the absence of any commercial or financial relationships that could be construed as a potential conflict of interest.

## Footnotes

^1^The choice of whitening or not the subject-level data changes the final group PCA estimates (Calhoun et al., 2015). However, all it does is preprocess the input data in a different way. While we advocate whitening of subject-level fMRI data for group spatial ICA due to its denoising properties, it remains optional and open for debate (Smith et al., 2014). However, whitening of subject-level data does not alter the correctness of the group PCA methods we present in this paper, which are correct regardless of the choice of preprocessing.

^2^http://mialab.mrn.org/software/gift/

^3^Direct reduction of the largest dimension of subject-level data is *never* recommended as it causes greater loss of information and largely inferior estimates than otherwise. Instead, a better approach is to carry out group PCA in the time dimension as in Figure 1, *without* subject-level whitening, followed by ${F_{i}^{T}=\Lambda^{-1∕2}X}^{T}Z_{i}$ to perform reduction of the voxel dimension. Group temporal ICA can then be carried out on the temporally concatenated $F_{i}^{T}.$

^4^Large data means *both* dimensions of the data are larger than 10000 (assuming the data is stored using double precision).

^5^This was in addition to subject-level whitening.

^6^Notice that the Krylov subspace size increases with larger block sizes, as well as the time to solve economy-size singular value decomposition for each iteration.

^7^MATLAB's *eigs*(·) function is based on the IRAM method to estimate desired eigenvectors and eigenvalues.

^8^http://www.netlib.org/lapack/

## References

[B1] Allen E. A. Erhardt E. B. Damaraju E. Gruner W. Segall J. M. Silva R. F.. (2011). A baseline for the multivariate comparison of resting-state networks. Front. Syst. Neurosci. 5:2. 10.3389/fnsys.2011.00002 21442040 PMC3051178

[B2] Beckmann C. F. Smith S. M. (2004). Probabilistic independent component analysis for functional magnetic resonance imaging. IEEE Trans. Med. Imaging 23, 137–152. 10.1109/TMI.2003.822821 14964560

[B3] Bell A. J. Sejnowski T. J. (1995). An information maximisation approach to blind separation and blind deconvolution. Neural Comput. 7, 1129–1159. 10.1162/neco.1995.7.6.1129 7584893

[B4] Biswal B. B. Mennes M. Zuo X.-N. Gohel S. Kelly C. Smith S. M.. (2010). Toward discovery science of human brain function. Proc. Natl. Acad. Sci. 107, 4734–4739. 10.1073/pnas.0911855107 20176931 PMC2842060

[B5] Boubela R. N. Kalcher K. Huf W. Kronnerwetter C. Filzmoser P. Moser E. (2013). Beyond noise: using temporal ICA to extract meaningful information from high-frequency fMRI signal fluctuations during rest. Front. Hum. Neurosci. 7:168. 10.3389/fnhum.2013.00168 23641208 PMC3640215

[B6] Brand M. (2003). Fast online SVD revisions for lightweight recommender systems, in 2003 SIAM International Conference on Data Mining, eds Barbara D. Kamath C. (San Francisco, CA: SIAM).

[B7] Calhoun V. D. Adali T. (2012). Multisubject independent component analysis of fMRI: a decade of intrinsic networks, default mode, and neurodiagnostic discovery. IEEE Rev. Biomed. Eng. 5, 60–73. 10.1109/RBME.2012.2211076 23231989 PMC4433055

[B8] Calhoun V. D. Adali T. Pearlson G. D. Pekar J. J. (2001). A method for making group inferences from functional MRI data using independent component analysis. Hum. Brain Mapp. 14, 140–151. 10.1002/hbm.1048 11559959 PMC6871952

[B9] Calhoun V. D. Egolf E. Rachakonda S. (2004). Group ICA of fMRI Toobox. Available online at: http://mialab.mrn.org/software/gift

[B10] Calhoun V. D. Silva R. F. Adali T. Rachakonda S. (2015). Comparison of PCA approaches for very large group ICA. Neuroimage 118, 662–666. 10.1016/j.neuroimage.2015.05.047 26021216 PMC4554805

[B11] Erhardt E. B. Rachakonda S. Bedrick E. J. Allen E. A. Adali T. Calhoun V. D. (2011). Comparison of multi-subject ICA methods for analysis of fMRI data. Hum. Brain Mapp. 32, 2075–2095. 10.1002/hbm.21170 21162045 PMC3117074

[B12] Feinberg D. A. Setsompop K. (2013). Ultra-fast MRI of the human brain with simultaneous multi-slice imaging. J. Magn. Reson. 229, 90–100. 10.1016/j.jmr.2013.02.002 23473893 PMC3793016

[B13] Feinberg D. A. Moeller S. Smith S. M. Auerbach E. Ramanna S. Glasser M. F.. (2010). Multiplexed echo planar imaging for Sub-Second Whole Brain FMRI and fast diffusion imaging. PLoS ONE 5:e15710. 10.1371/journal.pone.0015710 21187930 PMC3004955

[B14] Freire L. Roche A. Mangin J. F. (2002). What is the best similarity measure for motion correction in fMRI time series? IEEE Trans. Med. Imaging 21, 470–484. 10.1109/TMI.2002.1009383 12071618

[B15] Friston K. Holmes A. Worsley K. J. Poline J. P. Frith C. D. Frackowiak R. S. (1995). Statistical parametric maps in functional imaging: a general linear approach. Hum. Brain Mapp. 2, 189–210. 10.1002/hbm.460020402

[B16] Funk S. (2006). Netflix Update: Try This at Home. Available online at: http://sifter.org/~simon/journal/20061211.html (Accessed April 10, 2015).

[B17] Halko N. Martinsson P. G. Shkolnisky Y. Tygert M. (2011a). An algorithm for the principal component analysis of large data sets. SIAM J. Sci. Comput. 33, 2580–2594. 10.1137/100804139

[B18] Halko N. Martinsson P. G. Tropp J. A. (2011b). Finding structure with randomness: probabilistic algorithms for constructing approximate matrix decompositions. SIAM Rev. 53, 217–288. 10.1137/090771806

[B19] Himberg J. Hyvarinen A. Esposito F. (2004). Validating the independent components of neuroimaging time series via clustering and visualization. Neuroimage 22, 1214–1222. 10.1016/j.neuroimage.2004.03.027 15219593

[B20] Kuczynski J. Wozniakowski H. (1992). Estimating the largest eigenvalue by the power and lanczos algorithms with a random start. SIAM J. Matrix Anal. Appl. 13, 1094–1122. 10.1137/0613066

[B21] Lehoucq R. Sorensen D. Yang C. (1998). ARPACK Users' Guide: Solution of Large-Scale Eigenvalue Problems with Implicitly Restarted Arnoldi Methods. Philadelphia, PA: SIAM.

[B22] Lehoucq R. B. Sorensen D. C. (1996). Deflation techniques for an implicitly restarted Arnoldi iteration. SIAM J. Matrix Anal. Appl. 17, 789–821. 10.1137/S0895479895281484

[B23] Li Y. (2004). On incremental and robust subspace learning. Pattern Recognit. 37, 1509–1518. 10.1016/j.patcog.2003.11.010

[B24] Li Y.-O. Adali T. Calhoun V. D. (2007). Estimating the number of independent components for functional magnetic resonance imaging data. Hum. Brain Mapp. 28, 1251–1266. 10.1002/hbm.20359 17274023 PMC6871474

[B25] Ma S. Correa N. M. Li X. L. Eichele T. Calhoun V. D. Adali T. (2011). Automatic identification of functional clusters in fMRI data using spatial dependence. IEEE Trans. Biomed. Eng. 58, 3406–3417. 10.1109/TBME.2011.2167149 21900068 PMC3222740

[B26] Martinsson P.-G. Szlam A. Tygert M. (2010). Normalized power iterations for the computation of SVD, in Neural Information Processing Systems Workshop 2010. (Whistler, BC).

[B27] Mitliagkas I. Caramanis C. Jain P. (2013). Memory limited, streaming PCA, in Advances in Neural Information Processing Systems 26, eds Burges C. J. C. Bottou L. Welling M. Ghahramani Z. Weinberger K. Q. (Curran Associates, Inc), 2886–2894.

[B28] Recktenwald W. (2000). Introduction to Numerical Methods and MATLAB: Implementations and Applications. Upper Saddle River, NJ: Prentice Hall.

[B29] Roweis S. (1997). EM Algorithms for PCA and SPCA, in Neural Information Processing Systems (NIPS 1997), eds Jordan M. Kearns M. Solla S. (Denver, CO: The MIT Press).

[B30] Rutishauser H. (1970). Simultaneous iteration method for symmetric matrices. Num. Math. 16, 205–223. 10.1007/BF02219773

[B31] Saad Y. (2011). Numerical Methods for Large Eigenvalue Problems: Revised Edition. Philadelphia, PA: SIAM.

[B32] Setsompop K. Gagoski B. A. Polimeni J. R. Witzel T. Wedeen V. J. Wald L. L. (2012). Blipped-controlled aliasing in parallel imaging for simultaneous multislice echo planar imaging with reduced g-factor penalty. Magn. Reson. Med. 67, 1210–1224. 10.1002/mrm.23097 21858868 PMC3323676

[B33] Smith S. M. Hyvärinen A. Varoquaux G. Miller K. L. Beckmann C. F. (2014). Group-PCA for very large fMRI datasets. Neuroimage 101, 738–749. 10.1016/j.neuroimage.2014.07.051 25094018 PMC4289914

[B34] Smith S. M. Miller K. L. Moeller S. Xu J. Q. Auerbach E. J. Woolrich M. W.. (2012). Temporally-independent functional modes of spontaneous brain activity. Proc. Natl. Acad. Sci. U.S.A. 109, 3131–3136. 10.1073/pnas.1121329109 22323591 PMC3286957

[B35] Wang Z. Wang J. Calhoun V. D. Rao H. Detre J. A. Childress A. R. (2006). Strategies for reducing large fMRI data sets for ICA. Magn. Reson. Imaging 24, 591–596. 10.1016/j.mri.2005.12.013 16735180

