# Supplementary material for: Memory Efficient PCA Methods for Large Group ICA
Source: Front Neurosci. 2016 Feb 2;10:17. doi: 10.3389/fnins.2016.00017 (PMC4735350; doi:10.3389/fnins.2016.00017)
Supplement: Supplementary file 1 [file DataSheet1.docx]

# Appendix

## How to select the projecting subspace size ($\boldsymbol{l}$) for MPOWIT?

If we set the multiplier $l$ to 1, regular subspace iteration is performed. As shown in Figure 9, subspace iteration takes a lot of iterations to converge and, therefore, $l$ is usually set to a value greater than 1. The number of iterations required for convergence decreases with an increase in the value of $l$. However, a larger value of $l$ will increase the memory required to solve the PCA problem. We selected $l=5$ in all group-level PCA analyses using MPOWIT in this paper since this leads to a small memory burden in our rank $k$ PCA approximation. Depending on the computer memory resources available, a value of $l=10$ could be selected for very large group ICA studies to reduce the number of data loading operations when using the MPOWIT (un-stacked) approach.

| 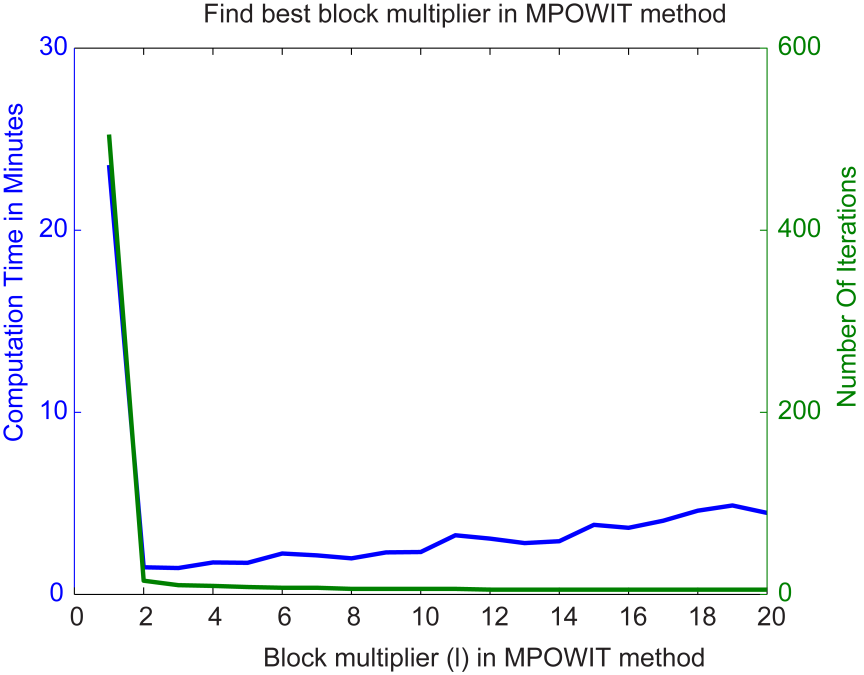  Figure 9: **Finding the best block multiplier for MPOWIT**. The block multiplier $l$ is varied from 1 to 20 to determine the best multiplier which helps the MPOWIT algorithm to converge in fewer number of iterations without additional burden on memory. We used a large temporally concatenated fMRI dataset of size 66745 by 40000 and extracted 100 components in this experiment. |
| --- |

## How much memory is required during the group-level PCA step?

Below, we give approximate memory requirements (in bytes) for each PCA algorithm. Let $Y$ be the temporally stacked data as mentioned in the Materials and Methods section with dimensions $v\times Mp$, where $M$ is the number of subjects, $p$ is the number of components retained in the first PCA step, and $v$ is the number of voxels. We assume that the variables are stored in double precision (8 bytes) and that the first step PCA was completed. In the following we use $k$ to denote the number of components extracted in the second PCA step.

**EVD**:

Typically, a copy of the variable $Y$ is stored in memory to compute $Y^{T}$, which is required to obtain the covariance matrix on the data. For large data analysis, in order to minimize the memory overhead of storing a copy of the variable in memory, only blocks of $Y^{T}$ (one at a time) are stored and used to compute the covariance matrix in the smallest dimension of the data. Eigenvectors resulting from the eigen-decomposition are projected onto the data. The memory required in bytes is given by:

$Memory=\left( vMp+min\left( v^{2},\left( Mp \right)^{2} \right)+k\cdot max\left( v,Mp \right)+k\cdot min\left( v,Mp \right) \right)\cdot8$. (38)

If the voxel dimension $v$ is smaller than the stacked time dimension $Mp$ (Equation (5)), the memory required in bytes is given by:

$Memory=\left( vp+v^{2}+v\cdot k+Mp\cdot k \right)\cdot8$. (39)

**Large PCA**:

Let $j$ be the number of block iterations and $b$ be the block length. The memory required by Large PCA for temporally stacked data is:

$Memory=\left( vMp+3\left( min\left( v,Mp \right)\cdot\left( j+1 \right)\cdot b \right)+\left( max\left( v,Mp \right)\cdot\left( j+1 \right)\cdot b \right) \right)\cdot8$. (40)

If each subject’s data is loaded one at a time (i.e., un-stacked), the memory required is given by:

$Memory=\left( vp+3\left( min\left( v,Mp \right)\cdot\left( j+1 \right)\cdot b \right)+\left( max\left( v,Mp \right)\cdot\left( j+1 \right)\cdot b \right) \right)\cdot8$. (41)

**MPOWIT**:

Let $l$ be the multiplier used to define the subspace (block) size. The memory required by MPOWIT for temporally stacked data is:

$\begin{matrix} M_{a}=2\left( \min\left( v,Mp \right)\cdot lk \right)+\left( lk \right)^{2} \\ M_{b}=min(v,Mp)\cdot lk+3(lk)^{2} \\ M_{c}=\min\left( v,Mp \right)\cdot k+\max\left( v,Mp \right)\cdot k \\ Memory=\left( vMp+\max\left( M_{a},M_{b},M_{c} \right) \right)\cdot8 \end{matrix}$ (42)

The memory computation in Equation (42) is divided into three parts:

1. $M_{a}$ - Computation of the covariance matrix in the smallest dimension.
2. $M_{b}$- Eigen-decomposition of the covariance matrix.
3. $M_{c}$- $F$ is obtained by projecting $X$ onto the data: $F=Y^{T}X$ (this step is required for back-reconstruction using GICA1).

From Equation (42), the memory required for the un-stacked version of MPOWIT is:

$Memory=\left( vp+max(M_{a},M_{b},M_{c}) \right)\cdot8$. (43)

Note that when back-reconstruction is not performed $M_{c}$ is not required.

**SVP**:

Let $lk$ be the number of components retained in the intermediate PCA step and $s$ be the subsampling depth selected. The memory required by SVP is given by:

$Memory=2\left( 2\left( \frac{v}{s^{3}} \right)^{2}+\left( \frac{v}{s^{3}} \right)\cdot\left( lk \right)+v\cdot\left( lk \right)+2\left( lk \right)^{2}+pk \right)\cdot8$. (44)

**STP**:

Let $lk$ be the number of components selected in the intermediate PCA step and $g$ is the number of subjects in each group. The memory required by STP is given by:

$Memory=\left( 2v\cdot\left( gp \right)+\left( gp \right)^{2}+2v\cdot\left( lk \right)+gp\cdot\left( lk \right)+4\left( lk \right)^{2} \right)\cdot8$. (45)

## Proof: MPOWIT and EM-PCA Converge to the Same PCA Subspace ($\boldsymbol{X}$)

Here we provide a proof that the algorithms presented for both MPOWIT and EM-PCA converge to the same PCA subspace estimate $X$ as long as both algorithms start from the same initial guess and execute the same number of iterations $j$. The proof is presented using mathematical induction, initially showing the base cases for $j=1$ and $j=2$ iterations, and then proving that if it holds for some $j\geq2$ then it must also hold for $j$ iterations.

Preamble:

Assume $Y\underset{\to}{SVD}XSF^{T}$ is the singular value decomposition of $Y$. Thus, $FS=Y^{T}X.$

If at iteration $j$, $X_{j}$ is the $j$-th orthonormal subspace estimate, then an approximation $\hat{X}$ can be obtained as follows: project the data onto $X_{j}$ as $Y^{T}X_{j}$; from SVD of $Y$ above $Y^{T}X_{j}=FSX^{T}X_{j}$ and ${X_{j}^{T}YY}^{T}X_{j}=$ ${X_{j}^{T}XS^{2}X}^{T}X_{j}$; from EVD $\frac{{X_{j}^{T}YY}^{T}X_{j}}{v-1}=W_{j}\Lambda_{j}W_{j}^{T}$ and $\Lambda^{-1/2}W_{j}^{T}\frac{{X_{j}^{T}YY}^{T}X_{j}}{v-1}W_{j}\Lambda^{-1/2}=I$; then an approximation of the eigenvectors is $F\approx Y^{T}\frac{X_{j}W_{j}\Lambda^{-1/2}}{\sqrt{v-1}}$. Thus, $\hat{X}={X_{j}W}_{j}\Lambda^{-1/2}$ so that $C_{\hat{X}}=\frac{\hat{X}^{T}\hat{X}}{v-1}=I$.

In the following, $k\ll v$.

Proof by induction:

Initialize with Gaussian samples ($j=0$): $X_{0}=G_{v\times k}$

A) Base cases:

$j=1$:

MPOWIT EM-PCA

$\boxed{\begin{aligned} \mathcal{X}_{0}=\left( YY^{T} \right)X_{0}\underset{\to}{SVD}UL\mathcal{F}^{T} \\ {{}} \\ \begin{matrix} \\ \end{matrix} \\ {} \\ orth\left( \mathcal{X}_{0} \right)\left\{ \begin{aligned} \begin{aligned} \mathcal{X}_{0}^{T}\mathcal{X}_{0}\mathcal{=F}LU^{T}UL\mathcal{F}^{T} \\ \mathcal{=F}L^{2}\mathcal{F}^{T} \\ X_{M1}=\left( YY^{T} \right)X_{0}\mathcal{F}L^{-1} \end{aligned} \\ =U \end{aligned} \right. \\ \frac{{X_{M1}^{T}YY}^{T}X_{M1}}{v-1}=W_{M1}\Lambda_{M1}W_{M1}^{T} \\ \hat{X}_{M}={X_{M1}W}_{M1}\Lambda_{M1}^{-1/2} \end{aligned}}$ $\boxed{\begin{aligned} F_{1}^{T}=X_{0}^{+}Y=\left( X_{0}^{T}X_{0} \right)^{-1}X_{0}^{T}Y \\ X_{1}=Y\left( F_{1}^{T} \right)^{+}=YF_{1}\left( F_{1}^{T}F_{1} \right)^{-1} \\ \begin{matrix} = \\ \end{matrix}\begin{matrix} \underbrace{YY^{T}X_{0}} \\ \mathcal{X}_{0} \end{matrix}\begin{matrix} \underbrace{\left( X_{0}^{T}YY^{T}X_{0} \right)^{-1}} \\ B \end{matrix}\begin{matrix} \left( X_{0}^{T}X_{0} \right) \\ {} \end{matrix} \\ X_{1}=\mathcal{X}_{0}B\left( X_{0}^{T}X_{0} \right)\underset{\to}{SVD}RSV^{T} \\ orth\left( X_{1} \right)\left\{ \begin{aligned} \begin{aligned} X_{1}^{T}X_{1}=VSR^{T}RSV^{T} \\ =VS^{2}V^{T} \\ X_{E1}=X_{1}VS^{-1} \end{aligned} \\ =R \end{aligned} \right. \\ \frac{{X_{E1}^{T}YY}^{T}X_{E1}}{v-1}=W_{E1}\Lambda_{E1}W_{E1}^{T} \\ \hat{X}_{E}={X_{E1}W}_{E1}\Lambda_{E1}^{-1/2} \end{aligned}}$

$${X_{E1}^{T}YY}^{T}X_{E1}\frac{1}{v-1}=S^{-1}V^{T}X_{1}^{T}YY^{T}X_{1}VS^{-1}\frac{1}{v-1}$$

$$=S^{-1}V^{T}\left( X_{0}^{T}X_{0} \right)B\mathcal{X}_{0}^{T}YY^{T}\mathcal{X}_{0}B\left( X_{0}^{T}X_{0} \right)VS^{-1}\frac{1}{v-1}$$

$$=S^{-1}V^{T}\left( X_{0}^{T}X_{0} \right)B\mathcal{F}LU^{T}YY^{T}UL\mathcal{F}^{T}B\left( X_{0}^{T}X_{0} \right)VS^{-1}\frac{1}{v-1}$$

$$=S^{-1}V^{T}\left( X_{0}^{T}X_{0} \right)B\mathcal{F}L\left( \frac{X_{M1}^{T}YY^{T}X_{M1}}{v-1} \right)L\mathcal{F}^{T}B\left( X_{0}^{T}X_{0} \right)VS^{-1}$$

$$\begin{matrix} = \\ \end{matrix}\begin{matrix} \underbrace{S^{-1}V^{T}\left( X_{0}^{T}X_{0} \right)B\mathcal{F}L} \\ A \end{matrix}\begin{matrix} W_{M1}\Lambda_{M1}W_{M1}^{T} \\ {} \end{matrix}\begin{matrix} \underbrace{L\mathcal{F}^{T}B\left( X_{0}^{T}X_{0} \right)VS^{-1}} \\ A^{T} \end{matrix}$$

$$A^{T}A=L\mathcal{F}^{T}B\left( X_{0}^{T}X_{0} \right)VS^{-1}S^{-1}V^{T}\left( X_{0}^{T}X_{0} \right)B\mathcal{F}L$$

$$=L\mathcal{F}^{T}B\left( X_{0}^{T}X_{0} \right)\left[ \left( X_{0}^{T}X_{0} \right)B\mathcal{X}_{0}^{T}\mathcal{X}_{0}B\left( X_{0}^{T}X_{0} \right) \right]^{-1}\left( X_{0}^{T}X_{0} \right)B\mathcal{F}L$$

$$=L\mathcal{F}^{T}B\left( X_{0}^{T}X_{0} \right)\left( X_{0}^{T}X_{0} \right)^{-1}B^{-1}\left( \mathcal{X}_{0}^{T}\mathcal{X}_{0} \right)^{-1}B^{-1}\left( X_{0}^{T}X_{0} \right)^{-1}\left( X_{0}^{T}X_{0} \right)B\mathcal{F}L$$

$$=L\mathcal{F}^{T}\left( \mathcal{X}_{0}^{T}\mathcal{X}_{0} \right)^{-1}\mathcal{F}L$$

$$=L\mathcal{F}^{T}\mathcal{F}L^{-2}\mathcal{F}^{T}\mathcal{F}L$$

$$=I$$

$$\Rightarrow A \text{is orthonormal}.$$

Thus, $\frac{{X_{E1}^{T}YY}^{T}X_{E1}}{v-1}=W_{E1}\Lambda_{E1}W_{E1}^{T}$ and $\frac{{X_{E1}^{T}YY}^{T}X_{E1}}{v-1}={AW}_{M1}\Lambda_{M1}W_{M1}^{T}A^{T}$; and since $A$ is orthonormal, it follows that $\Lambda_{E1}=\Lambda_{M1}$. Moreover, if all eigenvalues are distinct (like most cases in real data), it also follows that $W_{E1}=AW_{M1}$. When repeated eigenvalues are present, the EVD is not unique, particularly, the eigenvectors corresponding to repeated eigenvalues are not unique. However, practical EVD algorithms are deterministic in nature and consistently output the same set of eigenvectors. Therefore, so long as the EVD algorithm utilized for $\frac{{X_{E1}^{T}YY}^{T}X_{E1}}{v-1}$ is the same as the one utilized for $\frac{{X_{M1}^{T}YY}^{T}X_{M1}}{v-1}$, then $W_{E1}=AW_{M1}$. For clarity, notice that for a subset of $k$ repeated eigenvalues, the trailing $k-1$ eigenvectors are immediately determined from the orthogonality condition after the first eigenvector of the subset is chosen. In other words, the entire set is determined by the choice of the first of the $k$ eigenvectors. Finally,

$$\hat{X}_{E}=X_{E1}W_{E1}\Lambda_{E1}^{-\frac{1}{2}}$$

$$=\mathcal{X}_{0}B\left( X_{0}^{T}X_{0} \right)VS^{-1}AW_{M1}\Lambda_{M1}^{-\frac{1}{2}}$$

$$=\mathcal{X}_{0}B\left( X_{0}^{T}X_{0} \right)VS^{-1}S^{-1}V^{T}\left( X_{0}^{T}X_{0} \right)B\mathcal{F}LW_{M1}\Lambda_{M1}^{-\frac{1}{2}}$$

$$=\mathcal{X}_{0}B\left( X_{0}^{T}X_{0} \right)\left( X_{0}^{T}X_{0} \right)^{-1}B^{-1}\left( \mathcal{X}_{0}^{T}\mathcal{X}_{0} \right)^{-1}B^{-1}\left( X_{0}^{T}X_{0} \right)^{-1}\left( X_{0}^{T}X_{0} \right)B\mathcal{F}LW_{M1}\Lambda_{M1}^{-\frac{1}{2}}$$

$$=\mathcal{X}_{0}\left( \mathcal{X}_{0}^{T}\mathcal{X}_{0} \right)^{-1}\mathcal{F}LW_{M1}\Lambda_{M1}^{-\frac{1}{2}}$$

$$=\mathcal{X}_{0}\mathcal{F}L^{-2}\mathcal{F}^{T}\mathcal{F}LW_{M1}\Lambda_{M1}^{-\frac{1}{2}}$$

$$=\underset{X_{M1}}{\underbrace{\mathcal{X}_{0}\mathcal{F}L^{-1}}}W_{M1}\Lambda_{M1}^{-\frac{1}{2}}$$

$$=\hat{X}_{M}$$

This concludes base case $j=1$.

$j=2$:

MPOWIT EM-PCA

$\boxed{\begin{aligned} X_{1}=X_{M1} \\ \mathcal{X}_{1}=\left( YY^{T} \right)X_{M1}\underset{\to}{SVD}U_{1}L_{1}\mathcal{F}_{1}^{T} \\ {{}} \\ {} \\ {} \\ {{}} \\ {} \\ {} \\ {} \\ {} \\ orth\left( \mathcal{X}_{1} \right)\left\{ \begin{aligned} \mathcal{X}_{1}^{T}\mathcal{X}_{1}=\mathcal{F}_{1}L_{1}^{2}\mathcal{F}_{1}^{T} \\ X_{M2}=\left( YY^{T} \right)X_{M1}\mathcal{F}_{1}L_{1}^{-1} \\ =U_{1} \end{aligned} \right. \\ \frac{{X_{M2}^{T}YY}^{T}X_{M2}}{v-1}=W_{M2}\Lambda_{M2}W_{M2}^{T} \\ \hat{X}_{M}={X_{M2}W}_{M2}\Lambda_{M2}^{-1/2} \end{aligned}}$ $\boxed{\begin{aligned} X_{1}=RSV^{T}=X_{E1}SV^{T} \\ F_{2}^{T}=X_{1}^{+}Y=\left( X_{1}^{T}X_{1} \right)^{-1}X_{1}^{T}Y=VS^{-1}X_{E1}^{T}Y \\ X_{2}=Y\left( F_{2}^{T} \right)^{+}=YF_{2}\left( F_{2}^{T}F_{2} \right)^{-1} \\ =YY^{T}X_{E1}S^{-1}V^{T}\left( VS^{-1}X_{E1}^{T}YY^{T}X_{E1}S^{-1}V^{T} \right)^{-1} \\ =YY^{T}X_{E1}S^{-1}V^{T}VS\left( X_{E1}^{T}YY^{T}X_{E1} \right)^{-1}SV^{T} \\ =YY^{T}X_{E1}\frac{W_{E1}\Lambda_{E1}^{-1}W_{E1}^{T}}{v-1}SV^{T} \\ =YY^{T}X_{M1}\frac{W_{M1}\Lambda_{M1}^{-1}W_{M1}^{T}}{v-1}A^{T}SV^{T} \\ =YY^{T}X_{M1}\left( X_{M1}^{T}YY^{T}X_{M1} \right)^{-1}X_{M1}^{T}X_{E1}SV^{T} \\ =\underset{\mathcal{X}_{1}}{\underbrace{YY^{T}X_{M1}}}\underset{B_{1}}{\underbrace{\left( X_{M1}^{T}YY^{T}X_{M1} \right)^{-1}}}X_{M1}^{T}X_{1} \\ X_{2}=\mathcal{X}_{1}B_{1}\left( X_{M1}^{T}X_{1} \right)\underset{\to}{SVD}R_{2}S_{2}V_{2}^{T} \\ orth\left( X_{2} \right)\left\{ \begin{aligned} X_{2}^{T}X_{2}=V_{2}S_{2}^{2}V_{2}^{T} \\ X_{E2}=X_{2}V_{2}S_{2}^{-1} \\ =R_{2} \end{aligned} \right. \\ \frac{{X_{E2}^{T}YY}^{T}X_{E2}}{v-1}=W_{E2}\Lambda_{E2}W_{E2}^{T} \\ \hat{X}_{E}={X_{E2}W}_{E2}\Lambda_{E2}^{-1/2} \end{aligned}}$

$${X_{E2}^{T}YY}^{T}X_{E2}\frac{1}{v-1}=S_{2}^{-1}V_{2}^{T}X_{2}^{T}YY^{T}X_{2}V_{2}S_{2}^{-1}\frac{1}{v-1}$$

$$=S_{2}^{-1}V_{2}^{T}\left( X_{1}^{T}X_{M1} \right)B_{1}\mathcal{X}_{1}^{T}YY^{T}\mathcal{X}_{1}B_{1}\left( X_{M1}^{T}X_{1} \right)V_{2}S_{2}^{-1}\frac{1}{v-1}$$

$$=S_{2}^{-1}V_{2}^{T}\left( X_{1}^{T}X_{M1} \right)B_{1}\mathcal{F}_{1}L_{1}U_{1}^{T}YY^{T}U_{1}L_{1}\mathcal{F}_{1}^{T}B_{1}\left( X_{M1}^{T}X_{1} \right)V_{2}S_{2}^{-1}\frac{1}{v-1}$$

$$=S_{2}^{-1}V_{2}^{T}\left( X_{1}^{T}X_{M1} \right)B_{1}\mathcal{F}_{1}L_{1}\left( \frac{X_{M2}^{T}YY^{T}X_{M2}}{v-1} \right)L_{1}\mathcal{F}_{1}^{T}B_{1}\left( X_{M1}^{T}X_{1} \right)V_{2}S_{2}^{-1}$$

$$\begin{matrix} = \\ \end{matrix}\begin{matrix} \underbrace{S_{2}^{-1}V_{2}^{T}\left( X_{1}^{T}X_{M1} \right)B_{1}\mathcal{F}_{1}L_{1}} \\ A_{1} \end{matrix}\begin{matrix} W_{M2}\Lambda_{M2}W_{M2}^{T} \\ {} \end{matrix}\begin{matrix} \underbrace{L_{1}\mathcal{F}_{1}^{T}B_{1}\left( X_{M1}^{T}X_{1} \right)V_{2}S_{2}^{-1}} \\ A_{1}^{T} \end{matrix}$$

$$A_{1}^{T}A_{1}=L_{1}\mathcal{F}_{1}^{T}B_{1}\left( X_{M1}^{T}X_{1} \right)V_{2}S_{2}^{-1}S_{2}^{-1}V_{2}^{T}\left( X_{1}^{T}X_{M1} \right)B_{1}\mathcal{F}_{1}L_{1}$$

$$=L_{1}\mathcal{F}_{1}^{T}B_{1}\left( X_{M1}^{T}X_{1} \right)\left[ \left( X_{1}^{T}X_{M1} \right)B_{1}\mathcal{X}_{1}^{T}\mathcal{X}_{1}B_{1}\left( X_{M1}^{T}X_{1} \right) \right]^{-1}\left( X_{1}^{T}X_{M1} \right)B_{1}\mathcal{F}_{1}L_{1}$$

$$=L_{1}\mathcal{F}_{1}^{T}B_{1}\left( X_{M1}^{T}X_{1} \right)\left( X_{M1}^{T}X_{1} \right)^{-1}B_{1}^{-1}\left( \mathcal{X}_{1}^{T}\mathcal{X}_{1} \right)^{-1}B_{1}^{-1}\left( X_{1}^{T}X_{M1} \right)^{-1}\left( X_{1}^{T}X_{M1} \right)B_{1}\mathcal{F}_{1}L_{1}$$

$$=L_{1}\mathcal{F}_{1}^{T}\left( \mathcal{X}_{1}^{T}\mathcal{X}_{1} \right)^{-1}\mathcal{F}_{1}L_{1}$$

$$=L_{1}\mathcal{F}_{1}^{T}\mathcal{F}_{1}L_{1}^{-2}\mathcal{F}_{1}^{T}\mathcal{F}_{1}L_{1}$$

$$=I$$

$$\Rightarrow A_{1} \text{is orthonormal}.$$

Thus, $\frac{{X_{E2}^{T}YY}^{T}X_{E2}}{v-1}=W_{E2}\Lambda_{E2}W_{E2}^{T}$ and $\frac{{X_{E2}^{T}YY}^{T}X_{E2}}{v-1}=A_{1}W_{M2}\Lambda_{M2}W_{M2}^{T}A_{1}^{T}$; and since $A_{1}$ is orthonormal, it follows that $\Lambda_{E2}=\Lambda_{M2}$. Moreover, based on the discussion for $j=1$, it also follows that $W_{E2}=A_{1}W_{M2}$. Finally,

$$\hat{X}_{E}=X_{E2}W_{E2}\Lambda_{E2}^{-\frac{1}{2}}$$

$$=\mathcal{X}_{1}B_{1}\left( X_{M1}^{T}X_{1} \right)V_{2}S_{2}^{-1}A_{1}W_{M2}\Lambda_{M2}^{-\frac{1}{2}}$$

$$=\mathcal{X}_{1}B_{1}\left( X_{M1}^{T}X_{1} \right)V_{2}S_{2}^{-1}S_{2}^{-1}V_{2}^{T}\left( X_{1}^{T}X_{M1} \right)B_{1}\mathcal{F}_{1}L_{1}W_{M2}\Lambda_{M2}^{-\frac{1}{2}}$$

$$=\mathcal{X}_{1}B_{1}\left( X_{M1}^{T}X_{1} \right)\left( X_{M1}^{T}X_{1} \right)^{-1}B_{1}^{-1}\left( \mathcal{X}_{1}^{T}\mathcal{X}_{1} \right)^{-1}B_{1}^{-1}\left( X_{1}^{T}X_{M1} \right)^{-1}\left( X_{1}^{T}X_{M1} \right)B_{1}\mathcal{F}_{1}L_{1}W_{M2}\Lambda_{M2}^{-\frac{1}{2}}$$

$$=\mathcal{X}_{1}\left( \mathcal{X}_{1}^{T}\mathcal{X}_{1} \right)^{-1}\mathcal{F}_{1}L_{1}W_{M2}\Lambda_{M2}^{-\frac{1}{2}}$$

$$=\mathcal{X}_{1}\mathcal{F}_{1}L_{1}^{-2}\mathcal{F}_{1}^{T}\mathcal{F}_{1}L_{1}W_{M2}\Lambda_{M2}^{-\frac{1}{2}}$$

$$=\underset{X_{M2}}{\underbrace{\mathcal{X}_{1}\mathcal{F}_{1}L_{1}^{-1}}}W_{M2}\Lambda_{M2}^{-\frac{1}{2}}$$

$$=\hat{X}_{M}$$

This concludes base case $j=2$.

B) Inductive step:

MPOWIT EM-PCA

$\boxed{\begin{aligned} X_{j-1}=X_{M,j-1} \\ \mathcal{X}_{j-1}=\left( YY^{T} \right)X_{M,j-1}\underset{\to}{SVD}U_{j-1}L_{j-1}\mathcal{F}_{j-1}^{T} \\ {{}} \\ {} \\ {} \\ {{}} \\ {} \\ {} \\ \\ {} \\ {} \\ {} \\ orth\left( \mathcal{X}_{j-1} \right)\left\{ \begin{aligned} \mathcal{X}_{j-1}^{T}\mathcal{X}_{j-1}=\mathcal{F}_{j-1}L_{j-1}^{2}\mathcal{F}_{j-1}^{T} \\ X_{Mj}=\left( YY^{T} \right)X_{M,j-1}\mathcal{F}_{j-1}L_{j-1}^{-1} \\ =U_{j-1} \end{aligned} \right. \\ \frac{{X_{Mj}^{T}YY}^{T}X_{Mj}}{v-1}=W_{Mj}\Lambda_{Mj}W_{Mj}^{T} \\ \hat{X}_{M}={X_{Mj}W}_{Mj}\Lambda_{Mj}^{-1/2} \end{aligned}}$ $\boxed{\begin{aligned} X_{j-1}=X_{E,j-1}S_{j-1}V_{j-1}^{T} \\ F_{j}^{T}=X_{j-1}^{+}Y=\left( X_{j-1}^{T}X_{j-1} \right)^{-1}X_{j-1}^{T}Y=V_{j-1}S_{j-1}^{-1}X_{E,j-1}^{T}Y \\ X_{j}=Y\left( F_{j}^{T} \right)^{+}=YF_{j}\left( F_{j}^{T}F_{j} \right)^{-1} \\ =YY^{T}X_{E,j-1}S_{j-1}^{-1}V_{j-1}^{T}\left( V_{j-1}S_{j-1}^{-1}X_{E,j-1}^{T}YY^{T}X_{E,j-1}S_{j-1}^{-1}V_{j-1}^{T} \right)^{-1} \\ =YY^{T}X_{E,j-1}S_{j-1}^{-1}V_{j-1}^{T}V_{j-1}S_{j-1}\left( X_{E,j-1}^{T}YY^{T}X_{E,j-1} \right)^{-1}S_{j-1}V_{j-1}^{T} \\ =YY^{T}X_{E,j-1}\frac{W_{E,j-1}\Lambda_{E,j-1}^{-1}W_{E,j-1}^{T}}{v-1}S_{j-1}V_{j-1}^{T} \\ =YY^{T}X_{M,j-1}\frac{W_{M,j-1}\Lambda_{M,j-1}^{-1}W_{M,j-1}^{T}}{v-1}A_{j-1}^{T}S_{j-1}V_{j-1}^{T} \\ =YY^{T}X_{M,j-1}\left( X_{M,j-1}^{T}YY^{T}X_{M,j-1} \right)^{-1}X_{M,j-1}^{T}X_{E,j-1}S_{j-1}V_{j-1}^{T} \\ =\underset{\mathcal{X}_{j-1}}{\underbrace{YY^{T}X_{M,j-1}}}\underset{B_{j-1}}{\underbrace{\left( X_{M,j-1}^{T}YY^{T}X_{M,j-1} \right)^{-1}}}X_{M,j-1}^{T}X_{j-1} \\ X_{j}=\mathcal{X}_{j-1}B_{j-1}\left( X_{M,j-1}^{T}X_{j-1} \right)\underset{\to}{SVD}R_{j}S_{j}V_{j}^{T} \\ orth\left( X_{j} \right)\left\{ \begin{aligned} X_{j}^{T}X_{j}=V_{j}S_{j}^{2}V_{j}^{T} \\ X_{Ej}=X_{j}V_{j}S_{j}^{-1} \\ =R_{j} \end{aligned} \right. \\ \frac{{X_{Ej}^{T}YY}^{T}X_{Ej}}{v-1}=W_{Ej}\Lambda_{Ej}W_{Ej}^{T} \\ \hat{X}_{E}={X_{Ej}W}_{Ej}\Lambda_{Ej}^{-1/2} \end{aligned}}$

$${X_{Ej}^{T}YY}^{T}X_{Ej}\frac{1}{v-1}=S_{j}^{-1}V_{j}^{T}X_{j}^{T}YY^{T}X_{j}V_{j}S_{j}^{-1}\frac{1}{v-1}$$

$$=S_{j}^{-1}V_{j}^{T}\left( X_{j-1}^{T}X_{M,j-1} \right)B_{j-1}\mathcal{X}_{j-1}^{T}YY^{T}\mathcal{X}_{j-1}B_{j-1}\left( X_{M,j-1}^{T}X_{1} \right)V_{j}S_{j}^{-1}\frac{1}{v-1}$$

$$=S_{j}^{-1}V_{j}^{T}\left( X_{j-1}^{T}X_{M,j-1} \right)B_{j-1}\mathcal{F}_{j-1}L_{j-1}U_{j-1}^{T}YY^{T}U_{j-1}L_{j-1}\mathcal{F}_{j-1}^{T}B_{j-1}\left( X_{M,j-1}^{T}X_{1} \right)V_{j}S_{j}^{-1}\frac{1}{v-1}$$

$$=S_{j}^{-1}V_{j}^{T}\left( X_{j-1}^{T}X_{M,j-1} \right)B_{j-1}\mathcal{F}_{j-1}L_{j-1}\left( \frac{X_{Mj}^{T}YY^{T}X_{Mj}}{v-1} \right)L_{j-1}\mathcal{F}_{j-1}^{T}B_{j-1}\left( X_{M,j-1}^{T}X_{1} \right)V_{j}S_{j}^{-1}$$

$$\begin{matrix} = \\ \end{matrix}\begin{matrix} \underbrace{S_{j}^{-1}V_{j}^{T}\left( X_{j-1}^{T}X_{M,j-1} \right)B_{j-1}\mathcal{F}_{j-1}L_{j-1}} \\ A_{j-1} \end{matrix}\begin{matrix} W_{Mj}\Lambda_{Mj}W_{Mj}^{T} \\ {} \end{matrix}\begin{matrix} \underbrace{L_{j-1}\mathcal{F}_{j-1}^{T}B_{j-1}\left( X_{M,j-1}^{T}X_{1} \right)V_{j}S_{j}^{-1}} \\ A_{j-1}^{T} \end{matrix}$$

$$A_{j-1}^{T}A_{j-1}=L_{j-1}\mathcal{F}_{j-1}^{T}B_{j-1}\left( X_{M,j-1}^{T}X_{j-1} \right)V_{j}S_{j}^{-1}S_{j}^{-1}V_{j}^{T}\left( X_{j-1}^{T}X_{M,j-1} \right)B_{j-1}\mathcal{F}_{j-1}L_{j-1}$$

$$=L_{j-1}\mathcal{F}_{j-1}^{T}B_{j-1}\left( X_{M,j-1}^{T}X_{j-1} \right)\left[ \left( X_{j-1}^{T}X_{M,j-1} \right)B_{j-1}\mathcal{X}_{j-1}^{T}\mathcal{X}_{j-1}B_{j-1}\left( X_{M,j-1}^{T}X_{j-1} \right) \right]^{-1}\left( X_{j-1}^{T}X_{M,j-1} \right)B_{j-1}\mathcal{F}_{j-1}L_{j-1}$$

$$=L_{j-1}\mathcal{F}_{j-1}^{T}B_{j-1}\left( X_{M,j-1}^{T}X_{j-1} \right)\left( X_{M,j-1}^{T}X_{j-1} \right)^{-1}B_{j-1}^{-1}\left( \mathcal{X}_{j-1}^{T}\mathcal{X}_{j-1} \right)^{-1}B_{j-1}^{-1}\left( X_{j-1}^{T}X_{M,j-1} \right)^{-1}\left( X_{j-1}^{T}X_{M,j-1} \right)B_{j-1}\mathcal{F}_{j-1}L_{j-1}$$

$$=L_{j-1}\mathcal{F}_{j-1}^{T}\left( \mathcal{X}_{j-1}^{T}\mathcal{X}_{j-1} \right)^{-1}\mathcal{F}_{j-1}L_{j-1}$$

$$=L_{j-1}\mathcal{F}_{j-1}^{T}\mathcal{F}_{j-1}L_{j-1}^{-2}\mathcal{F}_{j-1}^{T}\mathcal{F}_{j-1}L_{j-1}$$

$$=I$$

$$\Rightarrow A_{j-1} \text{is orthonormal}.$$

Thus, $\frac{{X_{Ej}^{T}YY}^{T}X_{Ej}}{v-1}=W_{Ej}\Lambda_{Ej}W_{Ej}^{T}$ and $\frac{{X_{Ej}^{T}YY}^{T}X_{Ej}}{v-1}=A_{j-1}W_{Mj}\Lambda_{Mj}W_{Mj}^{T}A_{j-1}^{T}$; and since $A_{j-1}$ is orthonormal, it follows that $\Lambda_{Ej}=\Lambda_{Mj}$. Moreover, based on the discussion for $j=1$, it also follows that $W_{Ej}=A_{j-1}W_{Mj}$. Finally,

$$\hat{X}_{E}=X_{Ej}W_{Ej}\Lambda_{Ej}^{-\frac{1}{2}}$$

$$=\mathcal{X}_{j-1}B_{j-1}\left( X_{M,j-1}^{T}X_{j-1} \right)V_{j}S_{j}^{-1}A_{j-1}W_{Mj}\Lambda_{Mj}^{-\frac{1}{2}}$$

$$=\mathcal{X}_{j-1}B_{j-1}\left( X_{M,j-1}^{T}X_{j-1} \right)V_{j}S_{j}^{-1}S_{j}^{-1}V_{j}^{T}\left( X_{j-1}^{T}X_{M,j-1} \right)B_{j-1}\mathcal{F}_{j-1}L_{j-1}W_{Mj}\Lambda_{Mj}^{-\frac{1}{2}}$$

$$=\mathcal{X}_{j-1}B_{j-1}\left( X_{M,j-1}^{T}X_{j-1} \right)\left( X_{M,j-1}^{T}X_{j-1} \right)^{-1}B_{j-1}^{-1}\left( \mathcal{X}_{j-1}^{T}\mathcal{X}_{j-1} \right)^{-1}B_{j-1}^{-1}\left( X_{j-1}^{T}X_{M,j-1} \right)^{-1}\left( X_{j-1}^{T}X_{M,j-1} \right)B_{j-1}\mathcal{F}_{j-1}L_{j-1}W_{Mj}\Lambda_{Mj}^{-\frac{1}{2}}$$

$$=\mathcal{X}_{j-1}\left( \mathcal{X}_{j-1}^{T}\mathcal{X}_{j-1} \right)^{-1}\mathcal{F}_{j-1}L_{j-1}W_{Mj}\Lambda_{Mj}^{-\frac{1}{2}}$$

$$=\mathcal{X}_{j-1}\mathcal{F}_{j-1}L_{j-1}^{-2}\mathcal{F}_{j-1}^{T}\mathcal{F}_{j-1}L_{j-1}W_{Mj}\Lambda_{Mj}^{-\frac{1}{2}}$$

$$=\underset{X_{Mj}}{\underbrace{\mathcal{X}_{j-1}\mathcal{F}_{j-1}L_{j-1}^{-1}}}W_{Mj}\Lambda_{Mj}^{-\frac{1}{2}}$$

$$=\hat{X}_{M}$$

Therefore, $\hat{X}_{E}=\hat{X}_{M}$ if both run for the same number of iterations $j$ and use the same initial guess $X_{0}$.

## Parameter Selection for Large PCA

Here we study the optimality of Large PCA’s parameters $b$ and $j_{0}$ (see the section on Large PCA for a detailed description). We consider three different response measurements in our analysis: computation time in minutes, RAM memory used in GB, and number of dataloads. Since none of the three criteria alone suffices to establish preference of one parameter setting over the other, we considered all three simultaneously and determined the set of Pareto-optimal parameters based on the set of non-dominated points in the three-dimensional space of response measurements. The non-dominated points are those that cannot be outperformed simultaneously in all criteria by any other point. These are called Pareto-optimal and are indicated with a red circle in Figure 10 and Figure 11. The Pareto-optimal collection effectively outlines the trade-offs between each parameter setting. Points outside of the so-called Pareto-front are non-optimal since at least one method in the Pareto-front outperforms them in all criteria.

| 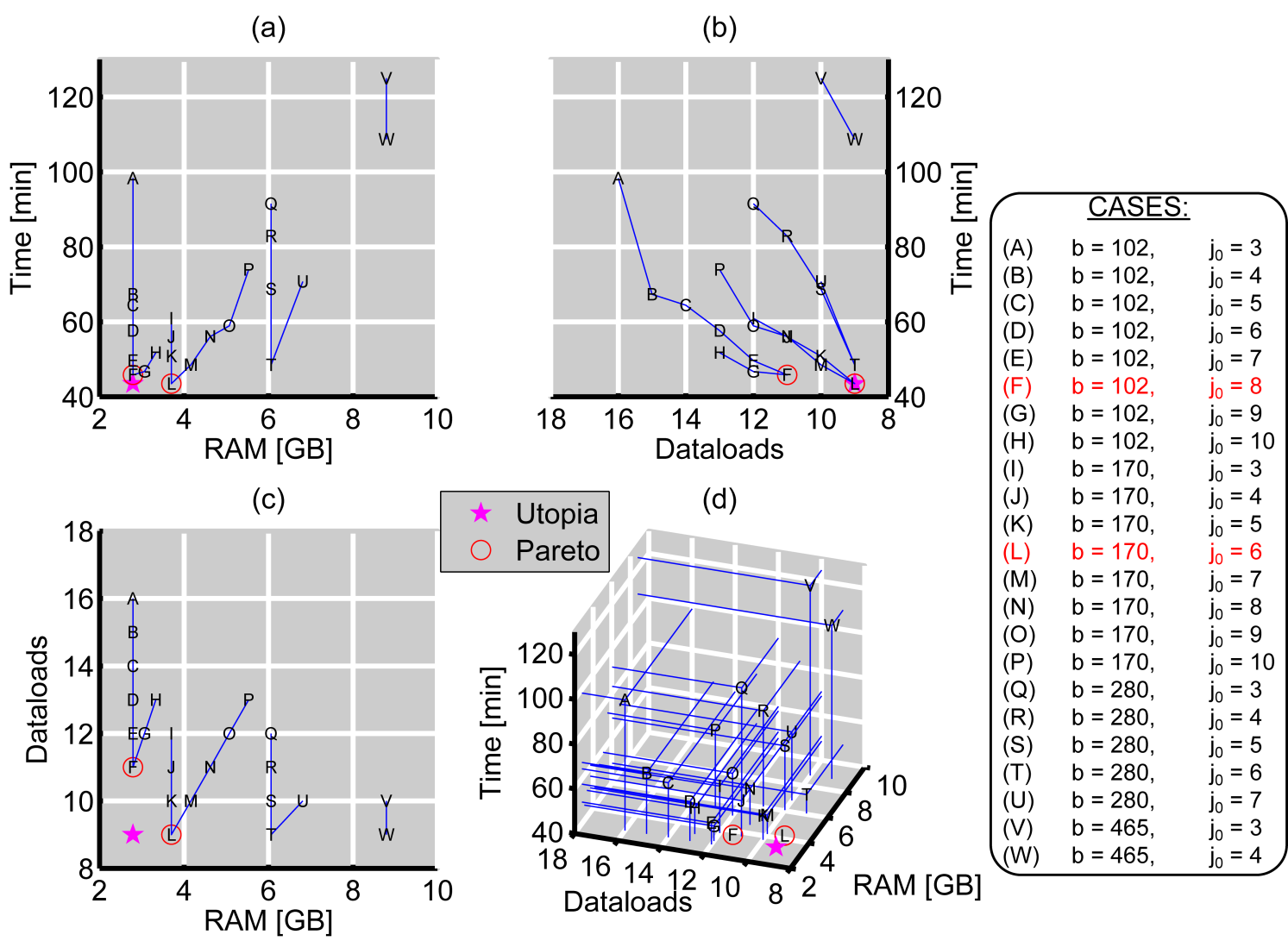  Figure 10: Pareto-optimal settings for 1600 real fMRI datasets on a Linux server. The utopia point combines the best performance measurements across all Pareto-optimal points. |
| --- |

| 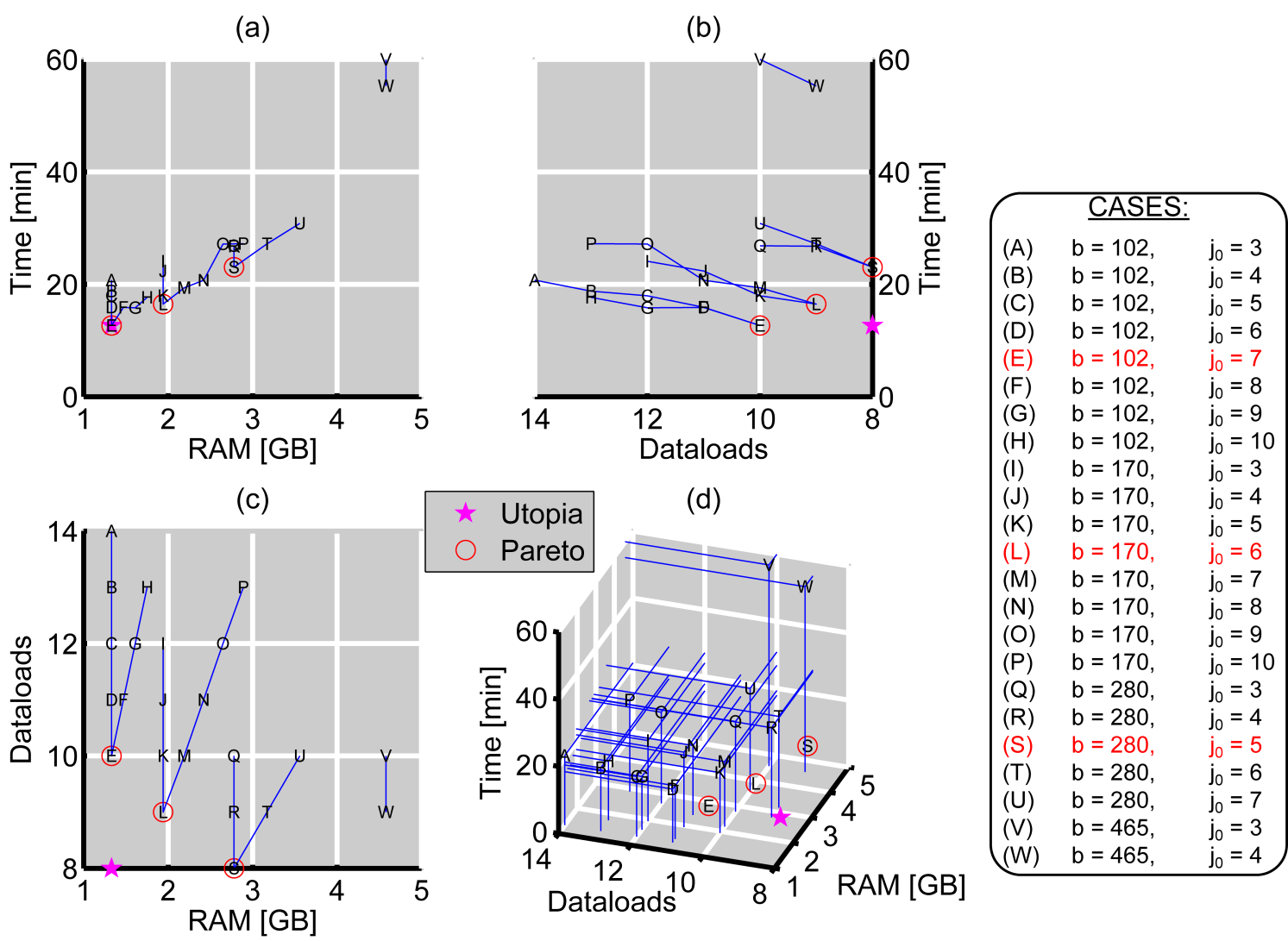  Figure 11: Pareto-optimal settings for 400 real fMRI datasets on a Linux server. The utopia point combines the best performance measurements across all Pareto-optimal points. |
| --- |

In practice, a parameter setting from the Pareto-front should be selected as a result of constraints from the problem at hand. For convenience, we select parameter setting L ($b=170$, $j_{0}=6$) since it is Pareto-optimal regardless of the dataset size. Note that the times reported here reflect mostly the time spent on arithmetic operations because the caching functionality of the server operating system retained the datasets in memory between iterations of Large PCA. The memory usage we report does not include the operating system cache. Clearly, Large PCA should be tuned to the datatype and computing system available to attain optimal performance.
